# *Candida tropicalis*-derived vitamin B3 exerts protective effects against intestinal inflammation by promoting IL-17A/IL-22-dependent epithelial barrier function

**DOI:** 10.1080/19490976.2024.2416922

**Published:** 2024-10-27

**Authors:** Ha T Doan, Li-Chieh Cheng, Yi-Ling Chiu, Yuan-Kai Cheng, Cheng-Chih Hsu, Yee-Chun Chen, Hsiu-Jung Lo, Hao-Sen Chiang

**Affiliations:** a Department of Life Science, National Taiwan University, Taipei, Taiwan; b Department of Chemistry, National Taiwan University, Taipei, Taiwan; c Leeuwenhoek Laboratories Co. Ltd, Taipei, Taiwan; d Department of Internal Medicine, National Taiwan University Hospital and National Taiwan University College of Medicine, Taipei, Taiwan; e National Institute of Infectious Disease and Vaccinology, National Health Research Institutes, Miaoli, Taiwan; f Genome and Systems Biology Degree Program, National Taiwan University, Taipei, Taiwan

**Keywords:** *Candida tropicalis*, inflammatory bowel disease, vitamin B3, IL-17A, IL-22, mucosal immunity, epithelial barrier function, epithelial cell proliferation

## Abstract

*Candida tropicalis-*a prevalent gut commensal fungus in healthy individuals – contributes to intestinal health and disease. However, how commensal *C. tropicalis* influences intestinal homeostasis and barrier function is poorly understood. Here, we demonstrated that the reference strain of *C. tropicalis* (MYA-3404) ameliorated intestinal inflammation in murine models of chemically induced colitis and bacterial infection. Intestinal colonization of *C. tropicalis* robustly upregulated the expression of IL-17A and IL-22 to increase barrier function and promote proliferation of intestinal epithelial cells in the mouse colon. Metabolomics analysis of fecal samples from mice colonized with *C. tropicalis* revealed alterations in vitamin B3 metabolism, promoting conversion of nicotinamide to nicotinic acid. Although nicotinamide worsened colitis, treatment with nicotinic acid alleviated disease symptoms and enhanced epithelial proliferation and Th17 cell differentiation. Oral gavage of *C. tropicalis* mitigated nicotinamide-induced intestinal dysfunction in experimental colitis. Blockade of nicotinic acid production with nicotinamidase inhibitors lowered the protective effects against colitis in mice treated with *C. tropicalis*. Notably, a clinical *C. tropicalis* strain isolated from patients with candidemia lacked the protective effects against murine colitis observed with the reference strain. Together, our results highlight a novel role for *C. tropicalis* in resolving intestinal inflammation through the modulation of vitamin B3 metabolism.

## SUMMARY

• Protection against colitis conferred by intestinal colonization of *Candida tropicalis* depends on metabolic activity and strain
• *C. tropicalis* MYA-3404 supplementation promotes intestinal epithelial barrier function through IL-17A and IL-22 expressed by Th17 cells, γδ T cells, and ILC3
• MYA-3404 strain uses its enzymatic activity to modulate vitamin B3 metabolism for protective benefits

## GRAPHICAL ABSTRACT

**Figure:**
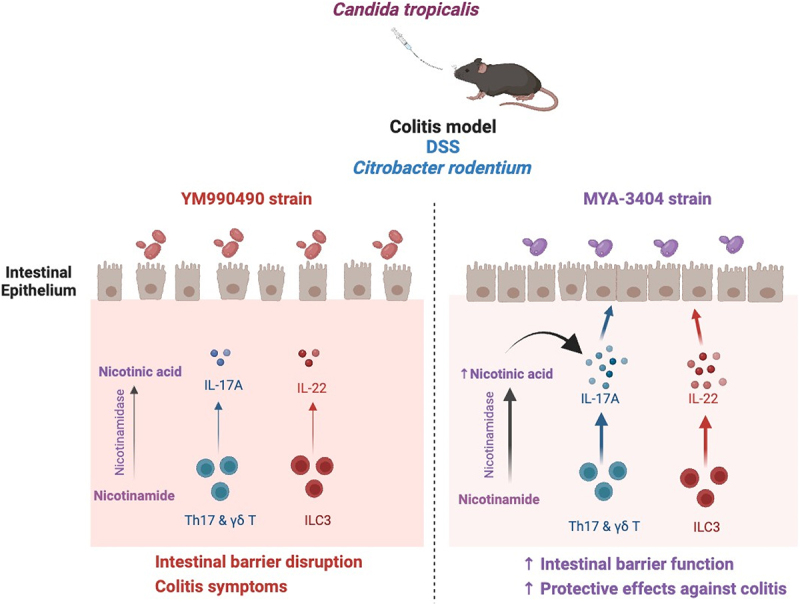

## Introduction

Inflammatory bowel disease (IBD), which includes Crohn’s disease (CD) and ulcerative colitis (UC), is characterized by persistent inflammation of the gastrointestinal tract and repeated cycles of relapse and remission.^1,2^ Rapidly increasing in prevalence worldwide, IBD negatively impacts patients’ quality of life and imposes significant social and economic costs.^3^ The etiology of IBD is multifactorial, involving interactions between environmental and genetic factors, gut microbiota, and dysregulated immune responses.^4,5^ Increasing evidence suggests that controlling the immune response through the gut microbiome is a practical approach to managing patients with IBD, further emphasizing the role of commensal microorganisms in IBD.^6,7^

Human gut microbiota comprises an intricate web of viruses, bacteria, fungi, and archaea.^8^ Studies on the significance of fungal species in the immunopathology of human diseases have been increasing.^9–11^ However, because fungal dysbiosis can cause gastrointestinal problems, it suggests that commensal fungi play a protective role in maintaining gut health.^12,13^ Numerous studies have shown the involvement of *Candida* spp. in the development of CD. The prevalence of *Candida tropicalis* increased in patients with CD compared with their healthy relatives.^14^ *Candida* spp. exacerbated intestinal inflammation through immune regulation and microbiome shifts.^10,15^ However, contrasting findings indicate that mucosa-associated fungi, including *Candida albicans*, *Saccharomyces cerevisiae*, and *Saccharomycopsis fibuligera*, can protect mice from colitis through the IL-22 pathway.^16^ Hence, the regulation of *Candida* spp. in response to inflammatory conditions appears to be context- and immune pathway-dependent.

Accumulating evidence underscores the importance of understanding the microbiome’s impact on host metabolism in elucidating its role in health and disease. Gut microbiota-derived metabolites, primarily from bacteria, such as short-chain fatty acids, tryptophan, and bile acid, are critical in maintaining immune homeostasis in IBD.^7,17^ Gut colonization by *S. cerevisiae* increased purine metabolism and uric acid production, disrupted the intestinal barrier, exacerbated illness, and increased intestinal permeability.^18^ However, it is unclear how gut microbiota protect against intestinal inflammation through metabolites and the immune system.

Although the barrier function of the intestinal epithelium and immunity ensures that luminal *Candida* spp. are separated from underlying tissues, commensal intestinal fungi can escape from this niche and reach the underlying lamina propria when the epithelial barrier is disrupted. The mechanism by which luminal fungi regulate intestinal barrier function during intestinal homeostasis and inflammation is unknown. In this study, we established a model of investing host – microbe interactions by administering broad-spectrum antibiotics for conventional mice followed by mono-colonization with a *C. tropicalis* reference strain or clinical strain. We found that supplementation of mice gut with the *C. tropicalis* reference strain (MYA-3404) protected mice against experimental colitis by promoting vitamin B3 metabolism via its nicotinamidase activity. The altered metabolites induced Th17- and γδ T-mediated IL-17A expression to enhance mucosal healing. Our findings suggest that *C. tropicalis* MYA-3404 has the unique ability to drive protective immunity with effects on intestinal barrier function and epithelial cell proliferation, as well as intestinal inflammation.

## Results

### Intestinal colonization of *C.* *tropicalis* alleviates dextran sulfate sodium-induced colitis dose dependently

Antibiotic administration profoundly affects the composition of the intestinal microbiome.^19^ Moreover, antibiotic exposure was associated with an increased risk of IBD at different ages.^20^ In mice with experimental colitis, *C. tropicalis* induced dysbiosis of commensal bacteria, leading to an enhanced proinflammatory phenotype.^15^ However, the direct effect of *C. tropicalis* on intestinal immune homeostasis and barrier function under antibiotic treatment remains unclear. Therefore, we asked whether colonization of mouse colon with limited intestinal commensal bacteria (antibiotic-treated) using *C. tropicalis* could exacerbate experimental colitis when intestinal integrity is damaged by dextran sulfate sodium (DSS). We selected *C. tropicalis* MYA-3404, which was developed from fingerprinting probes in 1996 and has the first published sequence with high similarity to the *C. albicans* genome.^21,22^ *C. tropicalis* MYA-3404 has been widely used as a reference strain to understand fungal behavior, such as susceptibility to antifungal drugs, and to evaluate the genetic, physiological, and metabolic characteristics of clinical and environmental isolates of fungi.^23,24^

**Figure 1.**
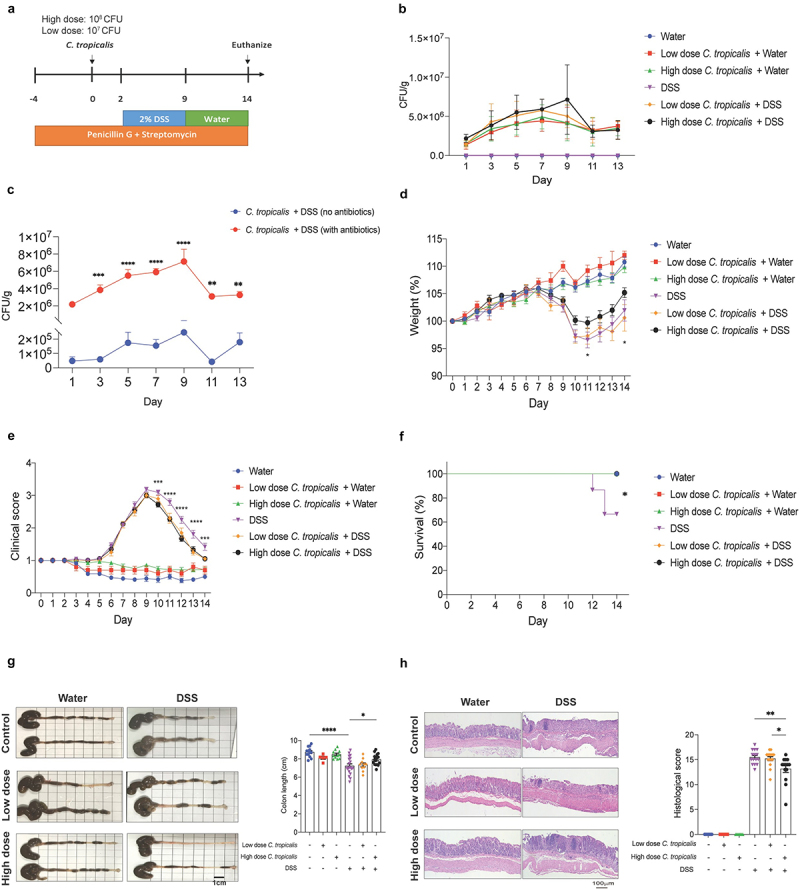
Oral supplementation of *Candida tropicalis* mitigates the severity of colitis during the resolution phase of intestinal inflammation.

To mimic the clinical niche in humans and ensure stable and prolonged colonization of *C. tropicalis* in the GI tract, we used cocktails of antibiotics, including streptomycin and penicillin, as described.^25^ After 4 d of pretreatment with antibiotics, mice were given oral gavage with two doses of *C. tropicalis*: high (10^8^ Colony formation unit (CFU)) and low (10^7^ CFU). We exposed the mice to 2% DSS for 7 d and subsequently replaced it with water for an additional 5 d to monitor the resolution phase of intestinal inflammation (Figure 1a). Comparable amounts of *C. tropicalis* were detected in the feces of mice among different groups throughout the experiment (Figure 1b). This result was in line with the finding that colonization of the GI tract with *Candida* spp. was independent of the inoculum dose.^26^ Supplementing water-treated mice with both doses of *C. tropicalis* maintained health conditions, aligning with the role of commensal fungi under homeostatic conditions. In addition, antibiotic administration markedly promoted *C. tropicalis* colonization in the gut (Figure 1c). Phenotypically, only high dose *C. tropicalis* supplementation group exhibited less pronounced weight loss (Figure 1d), but both doses showed significantly improved clinical scores (Figure 1e) compared with the DSS control group. Supplementation of both dosages of *C. tropicalis* profoundly prolonged survival in mice with DSS-induced colitis (Figure 1f). Compared to nonsupplemented mice treated with DSS, DSS-treated mice supplemented with a high dose of *C. tropicalis* exhibited a significant improvement in colon shortening – an indicator of DSS-induced inflammation severity (Figure 1g). Histological analysis revealed lower intestinal inflammation (less inflammatory infiltration cells, better-preserved crypts, and epithelial structures) in mice supplemented with a high dose of *C. tropicalis* than controls (Figure 1h). Interestingly, these protective effects against DSS-induced colitis were not found at the onset phase of inflammation (Figure S1b-d). Collectively, colonization of the mouse colon with *C. tropicalis* alleviated intestinal inflammation, particularly in the resolution phase, during experimental colitis in a dose-dependent manner.

(a) Graphical overview of experimental design. Colitis was induced by adding 2% DSS to drinking water for 7 d and switching back to water for 5 d. (b) Colony formation unit (CFU) of *C. tropicalis* in fecal samples every 2 d during the experiment. *n* = 10 per group from three experiments. (c) CFUs of *C. tropicalis* in fecal samples between two groups. *n* = 6–10 per group from three experiments. (d, e) Daily weight changes and total clinical scores of water- or DSS-treated mice with or without *C. tropicalis* supplementation. *n* = 10–20 per group from five experiments. (f) Percentage mice survival. *n* = 10 per *C. tropicalis* group, *n* = 15 per nonsupplementation group. Analyzed with the log-rank (Mantel – Cox) test. (g) Representative images and bar graphs of colon lengths. *n* = 7–20 per group from five experiments. (h) Representative H&E staining of the distal colon and total histopathology score. *n* = 5 per control group and *n* = 10 per DSS group from four experiments. Analyzed with two-way ANOVA followed by Turkey’s multiple comparisons test (Figure 1b-e), one-way ANOVA with Turkey’s multiple comparisons test (Figure 1g,h); *p < 0.05, **p < 0.01, ***p < 0.001, and ****p < 0.0001.

### *C.* *tropicalis* colonization induces IL-17A and IL-22 expression and increases intestinal barrier function and epithelial cell proliferation

**Figure 2.**
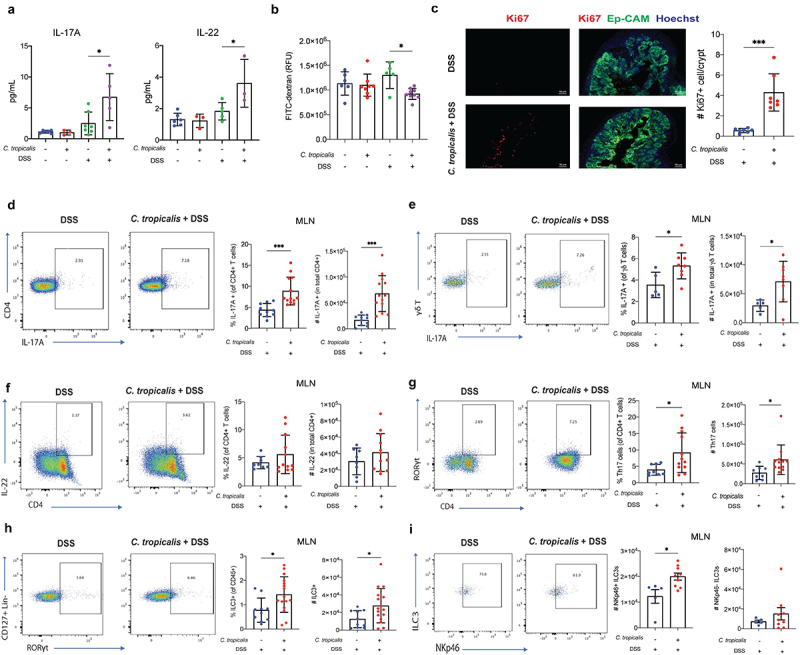
*Candida tropicalis* colonization in the gastrointestinal tract leads to the production of IL-17A by T cell subsets and IL-22 by ILC3s and reinforcement of intestinal barrier function.

Because fungal growth in the luminal part of the gut did not contribute to regulating colitis phenotypes, we investigated the host response to fungal colonization. Given that cytokines produced by the immune system play a critical role in the pathophysiology of IBD,^27^ we examined the cytokine profiles of colonic samples by multiplex assays. Interestingly, we observed a significant increase in IL-17A and IL-22 levels in the DSS-treated group supplemented with *C. tropicalis* (Figure 2a). The expression of IL-10 increased, whereas that of IL-23 and other proinflammatory cytokines was unchanged (Figure S2a). Because IL-17A and IL-22 are critical for maintaining intestinal barrier function,^28,29^ we asked whether *C. tropicalis* colonization modulates intestinal epithelial barrier function and integrity during colitis *in vivo*. On day 14, mice were orally gavaged with fluorescein isothiocyanate (FITC)-labeled dextran, and leakage of FITC-dextran in the mouse serum was monitored 4 h later to determine intestinal barrier permeability. Intestinal colonization of *C. tropicalis* significantly reduced recovery of FITC-dextran from the serum of DSS-treated mice (Figure 2b). Colitis-induced disturbance and deterioration of crypt structures subsequently reestablished during the healing phase through intestinal epithelial proliferation.^30^ To understand whether improved intestinal barrier function was due to increased proliferation of intestinal epithelial cells, we examined the level of Ki-67, a cell proliferation marker in Ep-CAM^+^ intestinal epithelial cells in the colon with fluorescence staining as Ep-CAM is a glycoprotein that is highly expressed in the intestinal epithelium and localized at the cell–cell junctions of the intestinal epithelium.^31–33^ Indeed, the colonization of *C. tropicalis* significantly induced the proliferation of colonic epithelial cells – indicated by an increase in Ki-67^+^ cells per intestinal crypt during the recovery phase (Figure 2c). Conversely, *C. tropicalis* supplementation did not induce epithelial cell proliferation during the induction phase of colitis (Figure S1f). Collectively, intestinal colonization with *C. tropicalis* enhanced IL-17A/IL-22 expression, barrier function, and epithelial cell proliferation in the colon of mice with DSS-induced colitis.

(a) IL-17A and IL-22 in the colonic homogenates of control and DSS-induced groups. The results are pooled data from two separate experiments. *n* = 3–7 per group. (b) FITC-dextran level in the serum 4 h after oral gavage. *n* = 5–9 per group from two experiments. (c) Representative immunofluorescence images of cell proliferation with the Ki-67 antibody (red). Epithelial cells are stained with Ep-CAM (green) and DNA with Hoechst (blue). Ki-67^+^ cells per crypt were quantified between control and *C. tropicalis* groups at 200×. *n* = 7 per group from two experiments. Scale bar = 50 μm. (d) Representative flow cytometry plots and quantification of IL-17A^+^ cells (percentage and absolute counts) from CD4^+^ T cells in MLNs of control and *C. tropicalis* groups. *n* = 10 in control, *n* = 13 in *C. tropicalis* group from four experiments. (e) Representative flow cytometry plots and quantification of IL-17A^+^ cells (percentage and absolute counts) from γδ T cells in the MLNs of control and *C. tropicalis* groups. *n* = 5 in control, *n* = 8 in *C. tropicalis* group from four experiments. (f) Representative flow cytometry plots and quantification of IL-22^+^ cells (percentage and absolute counts) from CD4^+^ T cells in the MLNs of control and *C. tropicalis* groups. *n* = 8 in control, *n* = 12 in *C. tropicalis* group from four experiments. (g) Representative flow cytometry plots and quantification of Th17 cells (percentage and absolute counts) from CD4^+^ T cells in the MLNs of control and *C. tropicalis* groups. *n* = 8 in control, *n* = 12 in *C. tropicalis* group from four experiments. (h) Representative flow cytometry plots and quantification of ILC3 cells (percentage and absolute counts) from CD45^+^ T cells in the MLNs of control and *C. tropicalis* groups. *n* = 10 in control, *n* = 16 in *C. tropicalis* group from four experiments. (i) Representative flow cytometry plots and absolute numbers of NKp46^±^ from ILC3s in the MLNs of control and *C. tropicalis* groups. *n* = 5 in the control group, *n* = 9 in the *C. tropicalis* group from two experiments. Statistical analyses were performed using one-way ANOVA (Figure 2a,b) and *t*-test (Figure 2c-i). *p < 0.05, **p < 0.01, and ***p < 0.001. Mesenteric lymph nodes (MLNs)

### Intestinal colonization of *C.* *tropicalis* stimulates the production of IL-17A by T cells and IL-22 by innate lymphoid cells type 3

To determine the cellular sources of IL-17A and IL-22, we isolated CD45^+^ immune cells from mesenteric lymph nodes (MLNs), followed by flow cytometry analysis, because MLNs are key sites for intestinal immunosurveillance and immune homeostasis during steady and inflammatory conditions.^34^ *C. tropicalis* supplementation significantly increased the percentage and total number of IL-17A^+^ CD4^+^ T cells and IL-17A^+^ γδ T cells in MLNs (Figure 2d,e), but had marginal effects on IL-22^+^ CD4^+^ T cells (Figure 2f). We found an increased percentage and number of Th17 cells in the *C. tropicalis*-supplemented group (Figure 2g). Furthermore, intestinal colonization of *C. tropicalis* significantly increased the population of innate lymphoid cells type 3 (ILC3) (Figure 2h). Because ILC3 induced both IL-17A and IL-22 production, we stained cells with anti-NKp46 antibodies to differentiate the source of these cytokines. NKp46^+^ ILC3 primarily generates IL-22, whereas NKp46^−^ ILC3 predominantly produces IL-17A.^35^ Notably, we observed a significant increase in NKp46^+^ ILC3 in the *C. tropicalis*-treated group (Figure 2i), indicating that ILC3 is the main origin of IL-22 expression. By contrast, our investigation did not reveal alterations in associated immune cell populations inside the lamina propria of colonic tissues (Figure S3a-e). A recent report found that T cell accumulation occurred earlier in MLN 14 d post-DSS treatment than colonic lamina propria (later time points on day 21).^36^ Our model assessed the immune cell population 12 d after DSS colitis onset, which could account for the significant change in MLN. Overall, intestinal colonization of *C. tropicalis* significantly induced IL-17A and IL-22 production by T cells (Th17 and γδ T cells) and ILC3, respectively, in MLNs.

### Protective effect of *C.* *tropicalis* against DSS-induced colitis is mediated by both IL-17A and IL-22

**Figure 3.**
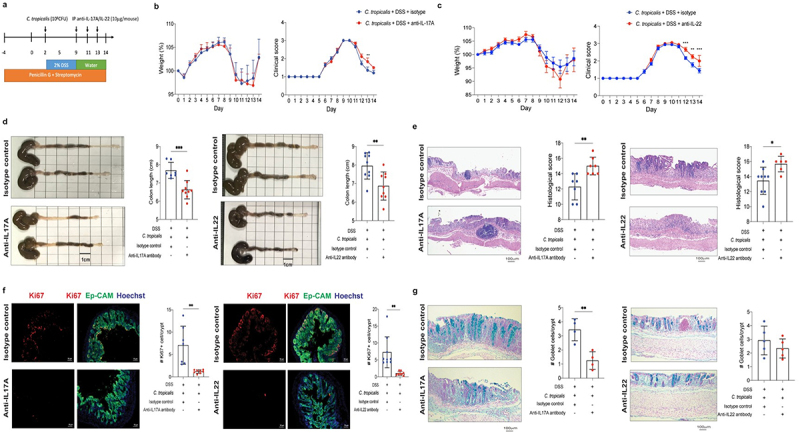
The protective function of *Candida tropicalis* against intestinal inflammation in mice is mediated by both IL-17A and IL-22.

We next asked if the observed phenotypes were mediated by IL-17A and IL-22 production in mice colonized with *C. tropicalis*. Because the increase in IL-17A and IL-22 was not observed during the induction phase of colitis (Figure S1e), we opted to administer neutralizing antibodies into mice during the recovery phase on days 9, 11, and 13 (Figure 3a). These neutralized antibodies effectively reduced cytokine expression in colonic samples (Figure S4). Interestingly, the functional depletion of both IL-17A and IL-22 exacerbated colitis but had minimal effect on weight loss (Figure 3b,c). For the assessment of intestinal inflammation, the administration of anti-IL-17A or anti-IL-22 neutralizing antibodies markedly shortened colon length and severely damaged intestinal structures in DSS-treated mice colonized with *C. tropicalis* (Figure 3d,e). DSS-treated mice receiving anti-IL-17A or anti-IL-22 neutralizing antibodies exhibited a considerable decrease in epithelial cell proliferation (Figure 3f). Because the loss of mucus-producing goblet cells is associated with DSS-induced colitis,^37^ we stained goblet cells using Alcian blue. Only IL-17A neutralization affected the number of goblet cells in the colon of *C. tropicalis*-supplemented DSS-treated mice (Figure 3g). In addition, recent studies have shed light into the contribution of the adaptive immune response, particularly secretory IgA binding to *Candida* spp. colonization, to intestinal inflammation.^38,39^ However, we did not detect changes in IgA in our model from different sources of secretory IgA binding to *C. tropicalis* in the feces, IgA^+^ cells in B cells, and IgA^+^ in plasma cells (Figure S5a-c). In summary, intestinal colonization of *C. tropicalis* mitigated intestinal inflammation depending on IL-17A and IL-22.

(a) Experimental design. Mice were intra-peritoneally injected with neutralizing antibodies on days 9, 11, and 13 of the recovery phase. (b) Weight loss and total clinical scores of mice injected with the isotype control and anti-IL-17A antibodies. *n* = 7–9 per group from two experiments. (c) Weight loss and total clinical scores of mice injected with the isotype control and anti-IL-22 antibodies. *n* = 9 per group from two experiments. (d) Representative image of the colon and colon lengths of mice injected with the isotype control and anti-IL-17A or anti-IL-22 antibodies. *n* = 7–9 per group from two experiments. (e) Representative image of H&E staining and histological scores of mice injected with the isotype control and anti-IL-17A or anti-IL-22 antibodies. *n* = 6–9 per group from two experiments. (f) Representative immunofluorescence images of cell proliferation with the Ki67 antibody. Quantification of the number of Ki-67^+^ cells per crypt between mice injected with the isotype control and anti-IL-17A or anti-IL-22 antibodies at 200×. *n* = 7–9 per group from two experiments. (g) Representative images of Alcian blue staining for goblet cells (blue color). Quantification of the number of goblet cells per crypt between the isotype control and anti-IL-17A or anti-IL22 antibodies at 200×. *n* = 4–5 per group from one experiment. Statistical analyses were analyzed with two-way ANOVA (Figure 3b,c) and *t*-test (Figure 3d-g). *p < 0.05, **p < 0.01, and ***p < 0.001.

### Alleviation of experimental colitis by *C.* *tropicalis* is metabolism- and strain-dependent

**Figure 4.**
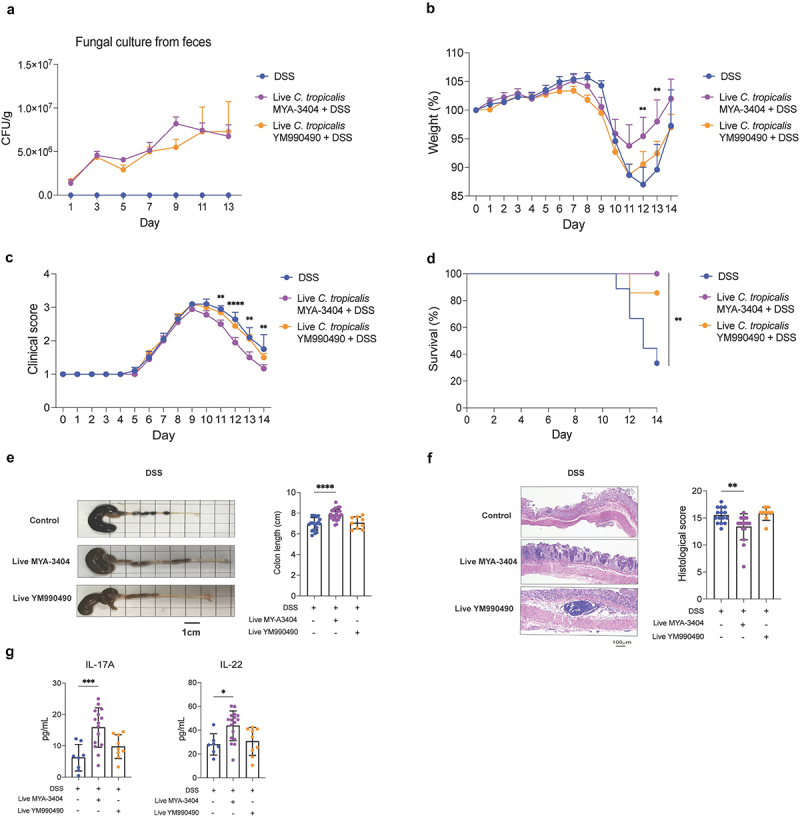
Supplementation of live *Candida tropicalis* MYA-3404 reference strain attenuates colitis severity.

In the gut, *S. cerevisiae* potentiated the production of metabolites that regulate the severity of intestinal inflammation.^18^ To determine whether DSS-induced colitis was alleviated due to metabolically active *C. tropicalis* MYA-3404 or a host reaction to fungal cell wall constituents, we continuously fed heat-killed *C. tropicalis* MYA-3404 to mice and subjected them to DSS-induced colitis (Figure S6a). Heat-killed MYA-3404 group showed no effects on colitis symptoms and intestinal inflammation (Figure S6b-d). To further delineate whether the protective effects against colitis of MYA-3404 were a universal signature across *C. tropicalis* strains, we included a clinically isolated *C. tropicalis* YM990490 strain from patients with candidemia in this setting.^40^ Both live *C. tropicalis* strains showed stable and comparable growth in the fecal samples throughout the experiment (Figure 4a). Live *C. tropicalis* MYA-3404 induced mild colitis with less body weight loss and improved colitis symptoms compared with uncolonized DSS controls, whereas live *C. tropicali*s YM990490 failed to alleviate colitis signatures (Figure 4b,c). Animals treated with live *C. tropicalis* MYA-3404 had improved survival (Figure 4d) and reduced intestinal inflammation, indicated by extended colon length (Figure 4e), maintenance of intestinal structures, and lower inflammatory cell infiltration into underlying layers (Figure 4f). By contrast, DSS controls and live YM990490 had similar phenotypes. We observed increased colonic expression of IL-17A and IL-22 that was specific to DSS-treated mice colonized with live MYA-3404 (Figure 4g), whereas IL-10 increased in mice colonized with MYA-3404 or YM990490 (Figure S2b). Importantly, these phenotypic changes of DSS-induced colitis initiated by live *C. tropicalis* MYA-3404 were not observed in mice colonized with live *C. tropicalis* YM990490, suggesting a strain-dependent effect of *C. tropicalis* MYA-3404. Since hyphal formation is critical for the induction of Th17 cells in *C. albicans* strains,^10^ we conducted an *in vitro* experiment to compare filamentation between MYA-3404 and YM990490. The results indicated that both MYA-3404 and YM990490 could induce hyphal forms; however, MYA-3404 was capable of producing more and faster-growing hyphae (Figure S7). In summary, the alleviation of intestinal inflammation during the recovery phase in DSS-treated mice colonized with *C. tropicalis* MYA-3404 did not simply reflect the host response to fungal components, but required a metabolically active organism in a strain-dependent manner.

(a) Fungal culture from fecal samples during the experiment (every 2 d). *n* = 3 per group from two experiments. (b) Body weight loss of colitis-induced mice with different strains of *C. tropicalis*. *n* = 9–20 per group from seven experiments. (c) Total clinical scores of DSS-treated mice with control and *C. tropicalis* groups. *n* = 9–20 per group from seven experiments. (d) Percentage survival in mice across six groups. Analyzed with the log-rank (Mantel – Cox) test. *n* = 9–20 per group from seven experiments. (e) Representative colon images and colon lengths of all groups. *n* = 9–20 per group from seven experiments. (f) Representative histopathological images of the distal colon and total histopathology scores. *n* = 9–20 per group from seven experiments. (g) Levels of IL-17A and IL-22 from the distal colon of different strains MYA-3404 and YM990490. *n* = 8–15 per group from three experiments. Statistical analyses were analyzed with two-way ANOVA (Figure 4a-c) and one-way ANOVA (Figure 4e-g). *p < 0.05, **p < 0.01, and ****p < 0.0001.

### *C.* *tropicalis* MYA-3404 colonization protects mice against bacterial infection

**Figure 5.**
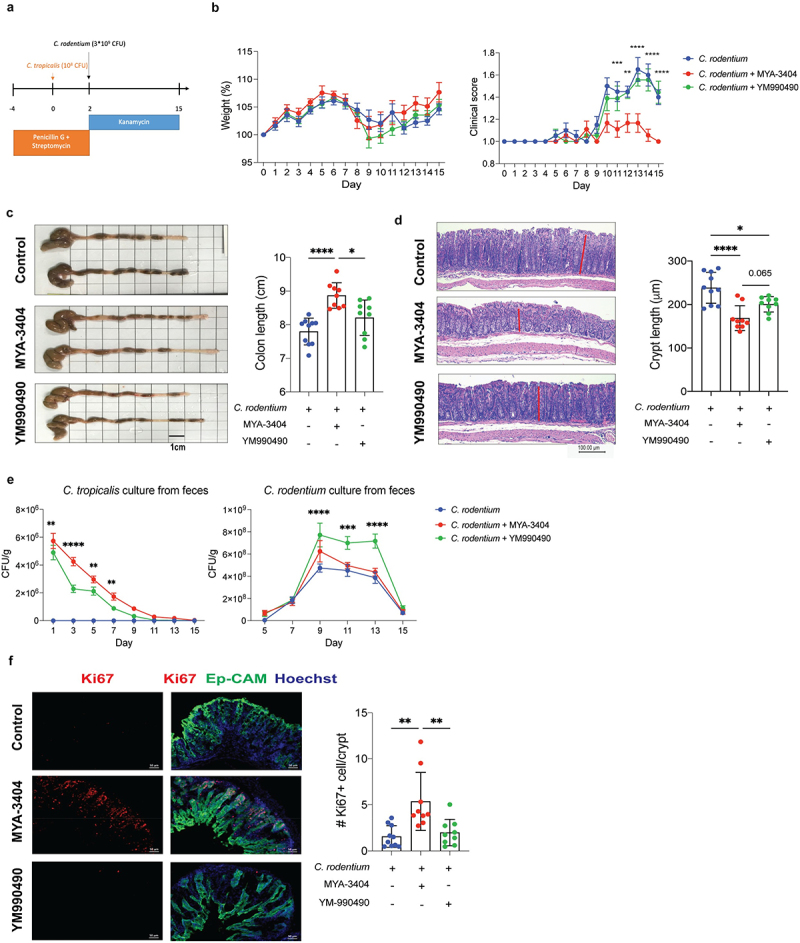
*Candida tropicalis* MYA-3404 facilitates the advantageous outcomes associated with bacterial infection through IL-17A and IL-22.

To independently validate the effects of *C. tropicalis* MYA-3404 on colitis severity, we used an alternative murine colitis model induced by *Citrobacter rodentium* infection. *C. rodentium* is a member of the attaching and effacing pathogens that damage intestinal epithelial cells, making it a valuable model for understanding IBD pathogenesis.^41,42^ Because we used a kanamycin-resistant *C. rodentium* strain, mice were administered kanamycin for stable bacterial colonization (Figure 5a).

(a) Experimental design. Colitis was induced by oral gavage with *C. rodentium* once and addition of kanamycin to drinking water for 13 d. (b) Daily weight loss and total clinical scores of control mice or mice infected with MYA-3404 and YM990490. *n* = 9–10 per group from two experiments. (c) Representative colon images and colon lengths of three groups. *n* = 9–10 per group from two experiments. (d) Representative images and crypt lengths of distal colons. Crypt length was measured as in red arrows. *n* = 9–10 per group from two experiments. (e) Fungal and bacterial cultures from fecal samples every 2 d during the experiment. *n* = 9–10 per group from two experiments. (f) Representative immunofluorescence images of cell proliferation with the Ki-67 antibody. The number of Ki-67^+^ cells per crypt across the three groups at 200×. *n* = 9–10 per group from two experiments. Statistical analyses were performed with two-way ANOVA (Figure 5b,e) and one-way ANOVA (Figure 5c,d,f). *p < 0.05, **p < 0.01, ***p < 0.001, and ****p < 0.0001.

Mice colonized with MYA-3404, but not YM990490, showed a significant reduction in colitis severity 13 d postinfection, although body weight loss remained consistent during bacterial infection (Figure 5b). MYA-3404 supplementation notably increased colon length (Figure 5c). Histopathological examination revealed nearly intact crypt and epithelial structures in the distal colon with mild invasive inflammatory cells. Thus, we did not employ the scoring system used in the DSS-induced model for evaluating *C. rodentium* infection. Instead, crypt hyperplasia, an important indicator of inflammatory severity, was assessed by measuring crypt length.^43^ Remarkably, the MYA-3404 group showed a significant reduction in crypt length, indicating lower crypt hyperplasia and intestinal inflammation than that in the uncolonized control group (Figure 5d). Subsequently, we assessed fungal and bacterial growth in the fecal samples. MYA-3404 showed a higher burden than YM990490 during the early phase of bacterial infection, with both strains witnessing a decrease in burden during the late stage. YM990490 exhibited a more abundant *C. rodentium* growth than the other two groups (Figure 5e). An increase in epithelial proliferation was associated with reduced average crypt length during inflammation.^30^ Indeed, MYA-3404 supplementation promoted epithelial cell proliferation within the crypt, whereas YM990490 failed to replicate this phenotype (Figure 5f).

The lack of variation in *C. rodentium* burden between control and MYA-3404 groups indicated that the protective effects of MYA-3404 on colitis were not due to a reduced number of *C. rodentium* in the intestine. We hypothesize that MYA-3404 colonization promotes the production of cytokines that regulate the host response to control *C. rodentium*-induced tissue damage. Cytokine expression analysis of colonic samples revealed increased IL-22 levels with MYA-3404 colonized in the gut (Figure S8a). We characterized IL-17A- and IL-22-related immune cell populations in MLNs. MYA-3404 inoculation increased the number of IL-17A^+^ CD4^+^ and IL-17A^+^ γδ T cells, whereas IL-22^+^ CD4^+^ T cells remained unchanged. We found a noticeable increase in NKp46^+^ ILC3^+^ cells, which are the primary source of IL-22 (Figure S8b). These immune cell alterations mirrored those observed in the chemical-induced colitis paradigm. Taken together, the intestinal colonization of MYA-3404 exhibited protective benefits against bacterial infection by promoting epithelial cell proliferation.

### *C.* *tropicalis* MYA-3404 colonization alters vitamin B3 metabolism that regulates epithelial proliferation and IL-17A production in a mouse colitis model

**Figure 6.**
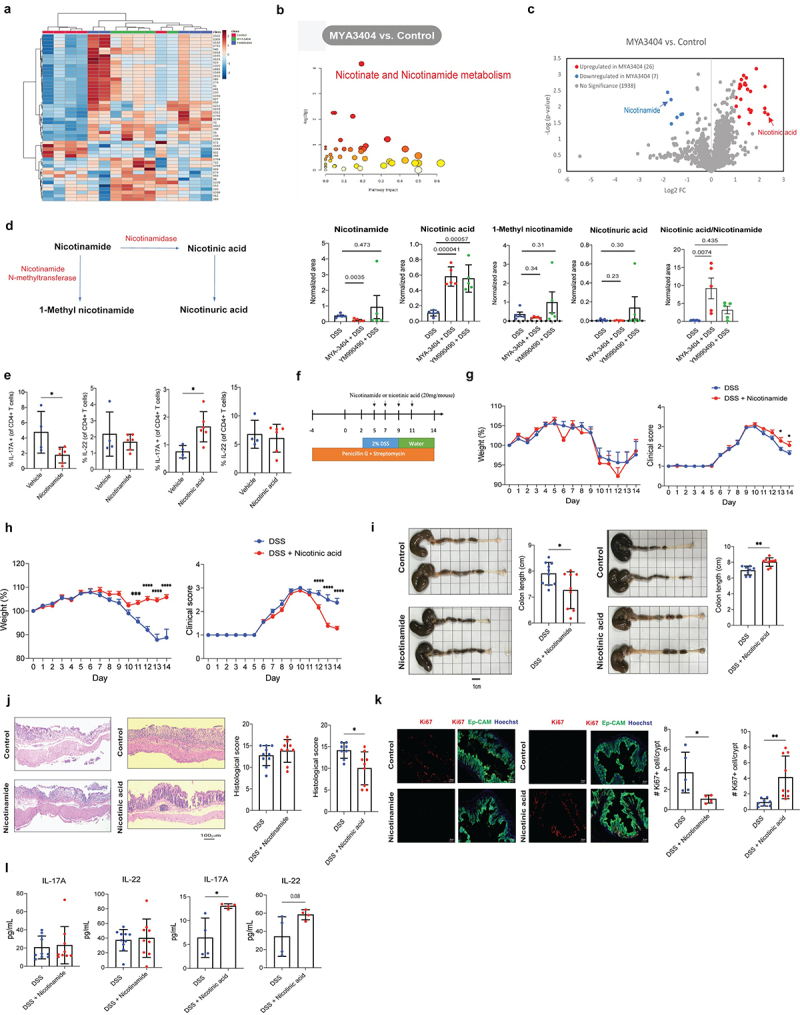
Intestinal colonization of *Candida tropicalis* MYA-3404 modulates vitamin B3 metabolism that regulates colitis severity and IL-17A expression.

Commensals produce endogenous metabolites from host-derived circulating components with their metabolic enzymes.^44^ Given that the metabolically active *C. tropicalis* MYA-3404 is required for improved intestinal inflammation in mice with experimental colitis, we hypothesize that live *C. tropicalis* MYA-3404 mediates these effects by influencing the gut metabolome. To support this hypothesis, we performed an untargeted metabolomics analysis from the feces of DSS-treated mice colonized with *C. tropicalis* MYA-3404 or YM990490. The fecal metabolomic profiles of mice colonized with MYA-3404 differed substantially from the metabolome of control mice or mice colonized with YM990490 (Figure 6a). Kyoto Encyclopedia of Genes and Genomes (KEGG) pathway analysis with MetaboAnalyst revealed that compared to control mice, nicotinate and nicotinamide metabolism was significantly upregulated in mice colonized with MYA-3404 and slightly changed in mice colonized with YM990490 (Figure 6b and Figure S9a). Nicotinate (also called nicotinic acid or niacin) and nicotinamide are known as vitamin B3, the precursors of coenzymes nicotinamide adenine dinucleotide (NAD) and nicotinamide adenine dinucleotide phosphate.^45–47^ Nicotinamide can be hydrolyzed to nicotinic acid by the microbial enzyme nicotinamidase.^48,49^ Interestingly, these two metabolites showed contrasting alterations in the MYA-3404 group. Specifically, nicotinamide was downregulated, whereas nicotinic acid was upregulated in the fecal metabolome (Figure 6c and Figure S9b). Mice colonized with MYA-3404, but not YM990490, had a significant increase in the nicotinic acid/nicotinamide ratio in the fecal metabolome. Both strains did not affect other metabolites in nicotinate and nicotinamide metabolism, including 1-methyl nicotinamide and nicotinuric acid (Figure 6d). Together, these results demonstrated that MYA-3404 colonization modulated vitamin B3 metabolism in mice during intestinal inflammation.

(a) Heatmap of measured metabolites in the feces. The graph shows the top 50 metabolite changes across the three groups. (b) Pathway analysis of differentially expressed metabolites was performed with MetaboAnalyst between MYA-3404 and control groups. (c) Volcano plot showing the fold change of metabolite quantity between MYA-3404 and control groups. Fold change > 2 or <−2, and *p*-value <0.05 are highlighted in red and blue, respectively. (d) Nicotinate and nicotinamide metabolism pathway (adapted from the KEGG database) and relative abundance of some metabolites involved in this pathway. (e) Naïve CD4^+^ T cells were cultured in the medium for 72 h with or without nicotinamide or nicotinic acid. Representative flow cytometry plots and percentages of IL-17A^+^ and IL-22^+^ cells from CD4^+^ T cells. *n* = 4–6 per group from two experiments. (f) Experimental design for oral gavage with nicotinamide and nicotinic acid on days 5, 7, 9, and 11. (g) Body weight change and total clinical scores of control mice and mice orally gavaged with nicotinamide. *n* = 9–10 per group from two experiments. (h) Body weight change and total clinical scores of control mice and mice orally gavaged with nicotinic acid. *n* = 8–9 per group from two experiments. (i) Representative images of the colon and measurement of colon length from control mice and mice orally gavaged with nicotinamide or nicotinic acid. *n* = 8–10 per group from two experiments. (j) Representative images of H&E staining and total histological scores of DSS control mice and mice administered nicotinamide or nicotinic acid. *n* = 8–10 per group from two experiments. (k) Representative immunofluorescence images of epithelial cell proliferation with the Ki-67 antibody from the distal colon. Quantification of the number of Ki-67^+^ cells per crypt between control mice and mice administered nicotinamide or nicotinic acid. *n* = 4–9 per group from two experiments. (l) IL-22 and IL-17A levels in the colon of control mice and mice administered nicotinamide or nicotinic acid. *n* = 4–10 per group from two experiments. Statistical analyses were performed with two-way ANOVA (Figures 6g,h), one-way ANOVA (Figure 6d), and *t*-test (Figure 6e,i-l). *p < 0.05, **p < 0.01, ***p < 0.001, and ****p < 0.0001.

Given that the intestinal colonization of *C. tropicalis* MYA-3404 increases the percentage of Th17 cells in MLNs, we examined whether nicotinamide or nicotinic acid differentially regulates Th17 differentiation *in vitro*. Although the frequency of IL-17A^+^ cells was significantly decreased when naïve CD4^+^ T cells were incubated with nicotinamide, a substantial increase in the frequency of IL-17A^+^ CD4^+^ cells was detected with nicotinic acid incubation. Neither metabolite altered the frequency of IL-22^+^ CD4^+^ cells in this setting (Figure 6e). To further determine the impact of nicotinamide and nicotinic acid on murine colitis *in vivo*, we subjected DSS-treated mice to each metabolite through oral gavage (Figure 6f). Animals administered nicotinamide had more signatures of intestinal inflammation, as indicated by increased clinical symptoms (Figure 6g) and colon shortening (Figure 6i). By contrast, oral supplementation of nicotinic acid profoundly improved the severity of colitis in mice with DSS-induced colitis (Figure 6h,i). Compared to control mice, mice with nicotinic acid administration had better histopathology, with preserved crypts and epithelial layers (Figure 6j). Consistently, nicotinic acid supplementation enhanced epithelial proliferation and IL-17A expression in the mouse colon (Figure 6k,l). We further defined the immune cell population in nicotinamide- or nicotinic acid-treated mice with colitis. Nicotinic acid treatments significantly increased IL-17A^+^ CD4^+^ T cells but not ILC3 in MLNs (Figure S9c), whereas nicotinamide supplementation inhibited the frequency of IL-17A^+^ CD4^+^ and IL-17A^+^ γδ T cells in the small intestine but not in MLNs (Figure S9c,d). Collectively, MYA-3404 intestinal colonization modulated intestinal inflammation, most likely through altered vitamin B3 metabolism (with increased conversion of nicotinamide to nicotinic acid), which promoted epithelial proliferation and IL-17A expression in the mouse colon.

### *C.* *tropicalis* MYA-3404 uses nicotinamidase activity to ameliorate nicotinamide-exacerbated intestinal inflammation

**Figure 7.**
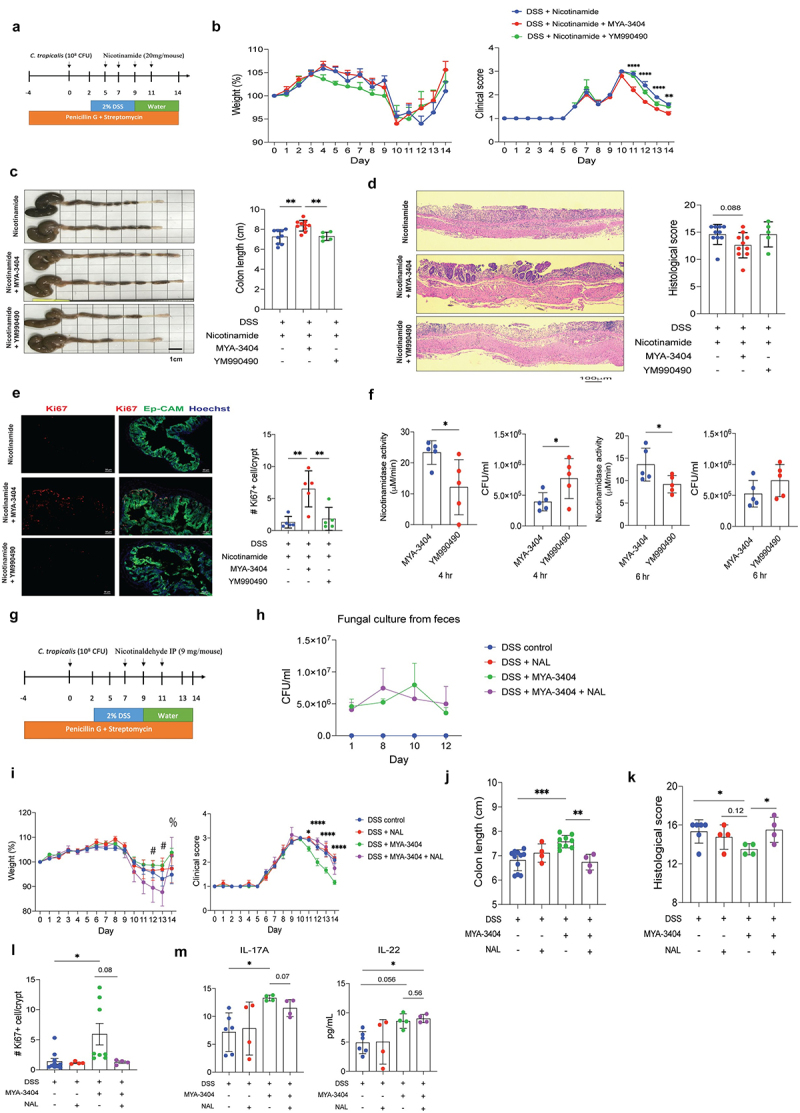
*Candida tropicalis* MYA-3404 utilizes nicotinamidase activity to ameliorate nicotinamide-exacerbated intestinal inflammation.

We have thus far shown that MYA-3404 colonization significantly increases the nicotinic acid/nicotinamide ratio in the fecal metabolome. We also found that although nicotinamide supplementation exacerbates intestinal inflammation in DSS-treated mice, oral gavage of nicotinic acid alleviates DSS-induced colitis. Therefore, we hypothesize that MYA-3404 colonization modulates experimental colitis in nicotinamide-administered mice. To examine the impact of *C. tropicalis* colonization on DSS-induced colitis *in vivo*, we supplemented nicotinamide-administered mice with MYA-3404 or YM990490 under DSS conditions (Figure 7a). MYA-3404 but not YM990490 colonization effectively mitigated colitis severity, including improved stool consistency, rectal bleeding, and colon shortening (Figure 7b,c) under nicotinamide treatment. MYA-3404 supplementation alleviated intestinal inflammation and enhanced epithelial cell proliferation in nicotinamide-treated mice with experimental colitis (Figure 7d,e). Thus, MYA-3404 colonization modulates intestinal inflammation potentially by altering nicotinamide metabolism in mice.

(a) Experimental design. Mice were orally gavaged with *C. tropicalis* and then gavaged with nicotinamide on days 5, 7, 9, and 11. (b) Body weight change and total clinical scores of mice with nicotinamide and *C. tropicalis*. *n* = 5–10 per group from two experiments. (c) Representative colon and colon length images from mice with nicotinamide and *C. tropicalis*. *n* = 5–10 per group from two experiments. (d) Representative images of H&E staining and total histological scores. *n* = 5–10 per group from two experiments. (e) Representative immunofluorescence images and number of Ki-67^+^ cells per crypt. *n* = 5 per group. (f) Nicotinamidase activity and fungal growth of *C. tropicalis* MYA-3404 and YM990490 strains *in vitro* after 4- and 6-h incubation. *n* = 4–5 per group from five independent experiments. (g) Experimental design. Mice were orally gavaged with *C. tropicalis* MYA-3404 and intraperitoneally injected with nicotinaldehyde three times. (h) Fungal culture from fecal samples across four groups. *n* = 3–4 per group from one experiment. Nicotinaldehyde (nicotinamidase inhibitor, NAL), (i) Weight change and clinical scores of four groups. *n* = 4–12 per group from two independent experiments (^#^*p* < 0.01, DSS + MYA-3404 vs. DSS + MYA-3404 + NAL; ^%^*p* < 0.001, DSS + MYA-3404 vs. DSS control; *p < 0.05, ****p < 0.0001, DSS + MYA-3404 vs. other three groups). (j) Colon length of all groups. *n* = 4–12 per group from two experiments. (k) Histopathological scores from the distal colon. *n* = 4–6 per group from one experiment. (l) Number of Ki67^+^ cells per crypt structure. *n* = 4–12 per group from two experiments. (m) IL-17A and IL-22 levels in colonic samples were determined with ELISA kits. *n* = 4–6 per group from one experiment. Statistical analyses were analyzed with two-way ANOVA (Figure 7b,h-i), one-way ANOVA (Figure 7c-e, j-m), and *t*-test (Figure 7f); *p < 0.05, **p < 0.01, ***p < 0.001, and ****p < 0.0001.

Nicotinamidase, encoded by *PNC1*, catalyzes the conversion of nicotinamide to nicotinic acid in the NAD^+^ salvage pathway, which is found in several microorganisms, including yeast, but is absent in mammals.^49,50^ Whole-genome sequencing data of MYA-3404 obtained from the National Center for Biotechnology Information database suggest that MYA-3404 contains a gene encoding nicotinamidase/pyrazinamidase (CTRG_05150). This finding implies that the MYA-3404 strain potentially uses this enzyme to modulate vitamin B3 metabolism for alleviating colitis.

To support this hypothesis, we set up a biochemical assay to examine nicotinamidase activity in MYA-3404 and YM990490 *in vitro*.^48^ MYA-3404 had significantly higher nicotinamidase activity than YM990490. Nicotinamide exhibited antifungal activity against *Candida* spp.^51^ The fungal CFU was even higher in YM99040 than MYA-3404, suggesting that changes in nicotinamidase activity were not due to different fungal growth capabilities (Figure 7f). Finally, we administered nicotinaldehyde, a potent nicotinamidase inhibitor,^49^ to mice colonized with MYA-3040 during DSS-induced colitis to evaluate the role of nicotinamidase in these experimental settings (Figure 7g). Treatment of the nicotinamidase inhibitor nicotinaldehyde did not affect fungal growth in the gut (Figure 7h). Notably, the administration of nicotinaldehyde significantly reversed the protective effects of MYA-3404 against colitis, indicated by worse symptoms of colitis, colon shortening, and intestinal inflammation (Figure 7i-k). Nicotinaldehyde reduced epithelial cell proliferation and IL-17A expression in the colon of DSS-treated mice colonized with MYA-3404 (Figure 7l,m). Collectively, *C. tropicalis* MYA-3404 uses nicotinamidase, at least partially, to alter nicotinamide metabolism, and thus, protect mice from experimental colitis by inducing IL-17A expression and promoting epithelial cell proliferation in the colon.

## Discussion

The benefits of commensal fungi have been increasingly recognized in maintaining intestinal homeostasis.^52^ However, our understanding of how this process is facilitated through the interaction between mycobiota, immune responses, and barrier function during intestinal inflammation is limited. In this study, we found that the stable colonization of *C. tropicalis-*the most abundant nonalbicans species in the human intestine – confers protection against injury to mucosal tissues and promotes the proliferation of epithelial cells during the recovery phase of colitis. These protective effects depend on IL-17A and IL-22 production. Using metabolomic analysis, we revealed an alteration of vitamin B3 metabolism in mice colonized with *C. tropicalis*. We demonstrated that *C. tropicalis* uses its enzymatic activities to promote the conversion of nicotinamide to nicotinic acid, which recapitulates protective effects against intestinal inflammation. By contrast, a clinical *C. tropicalis* strain isolated from patients with candidemia, who have decreased nicotinamidase activity, failed to confer the same protection against experimental colitis.

Several studies have provided insight into the involvement of *Candida* species in the development of IBD. The outgrowth of *Candida* spp. has been observed in the gastrointestinal tract of both UC and CD patients.^53–55^ Hoarau et al. reported a higher prevalence of *C. tropicalis* among CD patients compared to their healthy relatives.^14^ Notably, *C. tropicalis* aggravated the colitis in mice through Dectin-1 signaling.^12^ More recently, *C. tropicalis* isolated from IBD patients has been shown to exacerbate chronic intestinal inflammation in murine experimental colitis by inducing microbiome shifts.^15^ However, our study demonstrated a protective effect against DSS- and *C. rodentium*-induced colitis in mice treated with broad-spectrum antibiotic treatment, further emphasizing the direct impact of *C. tropicalis* on intestinal inflammation, particularly since patients are often prescribed antibiotic treatment early on. The finding that fungal dysbiosis can lead to gastrointestinal disturbances may support the hypothesis that fungi play a protective role in maintaining microbial balance in the gut. The influence of *C. tropicalis* on intestinal inflammation may depend on the specific context and act through different pathways. This could also be due to the presence of different *C. tropicalis* strains in the gut, as our data showed that clinical strain *C. tropicalis* YM990490 does not exhibit the same protective effects against experimental colitis as the reference strain *C. tropicalis* MYA3404.

Humans are constantly exposed to fungi that generally do not cause diseases in immunocompetent hosts. Diverse fungal populations colonize mucosal surfaces, with the most populated and varied types of genera found in the gastrointestinal tract.^56^ Patients with gastrointestinal disorders are often treated with broad-spectrum antibiotics to deplete pathogenic bacteria related to gut inflammation.^57^ The usage of antibiotics profoundly affects the microbiome composition in the intestine, resulting in an expansion of opportunistic gut commensal fungi.^58^ These fungi may affect certain physiological functions that were overlooked when commensal bacteria were abundant in the gut. The effect of fungal colonization in the intestinal tract depends on species and conditions.^16,18^ Using both reference and clinical strains of *C. tropicalis*, we identified diverse outcomes on colitis severity between these two strains. Only MYA-3404 provided protective effects against chemical-induced colitis and bacterial infection in murine models. Our findings offer a model to investigate the role of specific fungal strains in the immune response, intestinal barrier function, and homeostasis during both the onset and resolution phases of intestinal inflammation.

The intestinal epithelium constantly interacts with the immune cells in the underlying lamina propria. Several immune cells, including mononuclear phagocytes, T cells, and ILCs, play a crucial role in maintaining intestinal barrier function.^59^ Intestinal commensal fungi are a critical factor in regulating immune homeostasis and inflammation-associated pathologies.^60,61^ Mice colonized with mucosa-associated fungi, including *C. albicans*, *S. cerevisiae*, and *S. fibuligera*, induce an increase in Th17 cells in the lamina propria and MLNs.^16^ Similarly, we observed a significant increase in the frequency of Th17, γδ T cells, and ILC3 in the MLNs of mice colonized with *C. tropicalis*. We found that *C. tropicalis* is a potent inducer of Th17- and γδ T cell-derived IL-17A, as well as ILC3-derived IL-22. Although the impact of *C. tropicalis* colonization on the induction of Th17 responses is less pronounced compared to other studies, this may be attributed to the lower efficacy of individual strain colonization in inducing IL-17A^+^CD3^+^CD4^+^ or IL-22^+^CD3^+^CD4^+^ T cells compared to mucosal fungal consortium (MUC)-colonized mice.^16^ Both cytokines IL-17A and IL-22 promote intestinal epithelial cell proliferation, with IL-17A also inducing goblet cell differentiation, which enhances barrier function. Studies have suggested that Th17 cells, γδ T cells, ILC3s, IL-17A, and IL-22 are key elements of the immune response to intestinal fungal colonization.^62–65^ Indeed, the production of Th17, which is induced by *Candida* spp., may offer protective effects in the inflamed gut, enhancing barrier repair functions.^66^ ILC3s play a key role in initiating the induction of Th17 cells and in differentiating T cells. After DSS treatment, ILC3s primarily produce IL-22 rather than IL-17.^28,67^ Our data align with these findings, highlighting the beneficial roles of *C. tropicalis* induced IL-17A and IL-22 and the crosstalk between fungal intestinal colonization and host immune response in ameliorating colitis.

A recent study has shown that the hyphal morphogenesis program of *C. albicans* is critical for inducing Th17 responses in the murine intestine.^10^ The study also found that filamentation phenotypes vary among strains originating from human intestines. Although *C. tropicalis* MYA-3404 generated hyphae more rapidly and in greater quantities than YM990490 in our *in vitro* filamentation assay, we do not yet know how these strains alter their morphology within the mouse intestine, as the stimuli for hyphal formation differ *in vivo*. Additionally, the 2022 study by the Iliev group compared strains capable of hyphal formation, and non-filamentous isolates exhibit different effects on colitis, Th17 responses, and *in vitro* macrophage assays. Therefore, further experiments are needed to determine whether *Candida* strains with faster and more extensive hyphal growth are more effective at inducing colitis or Th17 responses *in vivo* compared to strains with slower and less extensive hyphal growth.

The crosstalk between intestinal commensals, immune responses, and intestinal barrier functions depends on microbial metabolites. The role of microbial metabolites in host health and pathophysiological function, particularly intestinal barrier function and immune responses, has been emphasized.^68^ Colonizing fungi actively secrete metabolites in the murine intestinal tract.^69^ Indeed, increased gut colonization of *S. cerevisiae* exacerbated colitis by increasing purine metabolism, leading to increased uric acid production.^18^ A study suggested that prostaglandin E2 produced by *C. albicans* could reach the lungs through circulation and promote allergic inflammation.^70^ Our experimental approach established a model for assessing the change of metabolic profiles of commensal fungi under conditions of gut inflammation. Although the protective effects against intestinal injury were mediated by mannan, a component of fungal cell walls,^71^ we did not notice significant improvement in intestinal inflammation when mice received heat-killed *C. tropicalis*. Our work rather suggests differences in metabolic profiles among commensal fungal strains. The results highlight the importance of intrinsic vitamin B3 metabolism by intestinal commensal fungi in modulating the immune response and severity of colitis. In addition to dietary intake, the gut microbiome serves as an essential source of vitamins.^72^ Our data suggest that *C. tropicalis* MYA-3404 uses its nicotinamidase to modulate vitamin B3 metabolism, which is in line with findings in commensal bacteria.^73^ Studies have indicated the beneficial effects of nicotinic acid on chemical-induced colitis by inhibiting proinflammatory pathways by acting on their factors or cytokine production.^74,75^ We explored an additional role of nicotinic in inducing IL-17A expression for promoting epithelium proliferation that ameliorates intestinal inflammation.

Fungi can be used as a probiotic treatment for inflammatory conditions.^76,77^ Supplementation of self-tunable yeast probiotics dampened intestinal damage by reducing fibrosis and dysbiosis.^77^ *Saccharomyces* spp. are used as safe probiotics for gastrointestinal disorders.^76^ Our results suggest a promising combined approach using vitamin B3 and *C. tropicalis* MYA-3404 for managing intestinal inflammation. Numerous *C. tropicalis* strains originate from various environmental, industrial, and clinical settings. Strains from different sources might exhibit distinct behaviors. Integrating genomic, transcriptomic, proteomic, and metabolic profiles, with host immune response during intestinal homeostasis and inflammation, will provide valuable insights into the mechanism and pathogenesis of these strains. This comprehensive approach can aid in developing novel therapies for colitis.

In conclusion, our findings enhance the understanding of how commensal fungi shape mucosal immunity through the host metabolome, thereby limiting intestinal inflammation.

## Limitations of the study

Several caveats in our study will need further investigation. We demonstrated that nicotinamide and nicotinic acid act on IL-17A^+^ cells, but the detailed mechanisms involved in how these molecules regulate immune cells need further exploration. The number of bacteria in the gut outnumbers fungi. Although we used antibiotics for the entire experiment, we cannot guarantee depletion of all bacterial populations. It is possible that *C. tropicalis* colonization in the intestine might impact bacterial growth. Similarly, some bacteria contribute to vitamin B3 metabolism enzymatically. Future studies will be necessary to identify the interkingdom interactions in the gut and the complexity of microbial symbiosis in regulating diseases through small molecules. This investigation focuses on a single species, and potential variations in metabolomes induced by other fungi were not examined.

## Materials and methods

### Mice

C57BL/6 female mice (6 weeks old) were purchased from the National Laboratory Animal Center (NARLAbs, Taiwan). The mice were kept and housed at the Animal Research Center of National Taiwan University (NTU) under Bio-safety 2 and maintained in a 12 h light – dark cycle with food and water *ad libitum*, according to the institutional guidelines. All experiments were conducted in accordance with approved protocols by the Institutional Animal Care and Use Committee at NTU (approval # NTU-110-041).

### DSS-induced colitis

In total, 2% DSS (36,000–50,000 Da, MP Biomedicals) was provided *ad libitum* in drinking water for 7 d. Then, water was provided for a recovery period of 5 d. Colitis symptoms were checked daily by measuring weight loss and total clinical scores by summing stool consistency and bleeding in the stool.^37^

### Metabolite supplementation

In total, 20 mg nicotinamide (N0636; Sigma-Aldrich) and 20 mg nicotinic acid (N0761; Sigma-Aldrich) were dissolved in 150 μL water and 1 M NaOH, respectively. For the vehicle, we used the respective buffers. Mice were oral gavaged with 20 mg metabolite per mouse each time.

For the nicotinamidase inhibitor, the mice were intraperitoneally injected with 9 mg nicotinaldehyde (P62208; Sigma-Aldrich) in 100 μL water each time.

### Fungal supplementation

*C. tropicalis* MYA-3404 and YM-990490 from patients with candidemia (kindly provided by Dr Hsiu-Jung Lo, National Health Research Institutes, Taiwan) were used in this study. *C. tropicalis* was cultured in Yeast Extract-Peptone-Dextrose (YPD: 20 g/L peptone, 10 g/L yeast extract, and 2% glucose) broth at 30°C with shaking at 200 rpm overnight. The broth culture was centrifuged for 5 min at 3,000 rpm and washed once with sterile PBS. One mouse was orally gavaged with 10^8^ or 10^7^ CFU *C. tropicalis* in 100 μL PBS. Fecal pellets were collected 1 d after fungal inoculation to assess fungal colonization.

For the heat-killed form of *C. tropicalis*, 10^8^ CFU/mL was incubated in a water bath at 70°C for 1 h. The heat-killed form was streaked on YPD plates (20 g/L peptone, 10 g/L yeast extract, 2% glucose, and 20 g/L agar) and incubated for 48 h at 30°C to confirm no growth. Mice were oral gavage with heat-killed form in 100 μL PBS every day.

### Determination of fungal burden

Feces were collected, weighed, and mixed with sterile PBS to yield 100 mg/mL. Samples were spun down. The supernatant was serially diluted and cultured on YPD agar plates supplemented with gentamycin (10 μg/mL; 101-1405-41-0; Cyrus Bioscience) and ampicillin (50 μg/mL; 101-69-52-3; MDBio). Plates were incubated for 24 h at 30°C before reading out the number of colonies. The result is presented as CFU/g.

### *Citrobacter rodentium* infection model

A kanamycin-resistant strain of *C. rodentium* was gifted by Dr. Jr-Wen Shui (Academia Sinica, Taiwan). *C. rodentium* was cultured on Luria-Bertani (LB) plates (10 g/L tryptone, 5 g/L yeast extract, 10 g/L sodium chloride, and 15 g/L agar). A single colony was incubated in 3 mL LB broth (10 g/L tryptone, 5 g/L yeast extract, 10 g/L sodium chloride) with 50 μg/mL kanamycin (101-25,389-94-0; Cyrus Bioscience) at 37°C for 16 h with shaking at 200 rpm until OD600 was 1.0 (approximately 1.2 × 10^9^ CFU/mL). The desired number of CFU was calculated and washed in sterile PBS and centrifuged for 10 min at 3,000 × *g* at 4°C. Mice were inoculated with 3 × 10^9^ CFU in 100 μL PBS. Body weight, stool consistency, and blood in stool were checked daily. Feces were collected, weighed, and diluted for culture on LB plates with 50 μg/mL kanamycin to quantify *C. rodentium* growth.

### Antibiotic treatment for animals

A cocktail of 2 mg/mL streptomycin (STP101.50; BioShop) and 1,500 U/mL penicillin G (PEN333.1; BioShop) was added to drinking water for the whole experiment.^78^ The animals were monitored daily, and antibiotics were replaced every 3 d.

### Neutralizing antibody injection

Mice were injected through intraperitoneal (IP) with 10 μg isotype control (16-4714-85; Thermo Fisher Scientific) or anti-IL17A (16-7173-85; Thermo Fisher Scientific) and 10 μg isotype control (16-4321-85; Thermo Fisher Scientific) or anti-IL22 antibodies (16-7222-85; Thermo Fisher Scientific) every other day during the recovery phase of the experiment (on days 9, 11, and 13 post-fungal infection).

### Histopathological analysis

Distal colon tissue was fixed in 10% formaldehyde solution, embedded in paraffin, and stained with hematoxylin and eosin or Alcian blue. Tissue sections were cut and stained by a blinded and professional pathologist, according to a scoring system.^79^ Five parameters (inflammatory severity, infiltration of inflammatory cells, tissue regeneration, crypt damage, and involved percentage) were used for histological assessment. The histological score was the sum of each score (maximum score: 18).

For *C. rodentium* infection evaluation, crypt hyperplasia was measured with the elongation of colonic crypts by measuring the length of at least 20 well-oriented crypts surrounded by inflammatory cells, as described.^80^

For Alcian blue staining, goblet cells were stained with blue, and the number of goblet cells per crypt was determined.

### Cytokine measurement

Distal colon tissue was homogenized in 1% NP40/PBS (NON 505.100; BioShop) and centrifuged at 10,000 rpm for 20 min to collect the supernatant. Protein concentration was determined with a BCA kit (PI23227; Thermo Fisher Scientific). A total of 50 μg protein was used as input for enzyme-linked immunosorbent assays (ELISA). IL-17A (88-7371-86; Invitrogen) and IL-22 (DY582-05; R&D Systems) kits were used according to the manufacturer’s instructions, with a minor modification (samples were incubated overnight at 4°C).

We used Multi-Plex Immunoassay (using a bead set coated with capture antibodies) for panels of IL-17A, IL-22, IL-10, IL-6, IL-23, IL-1β, TNF-α, and IFN-γ of colonic homogenates.

### Isolation of immune cells from MLN and intestinal lamina propria

Lamina propria were isolated from whole large intestine and small intestine (7 cm of terminal ileum), as described, with some modifications.^10,81^ In brief, the intestine was opened, the luminal content was washed twice with PBS, and the intestine was cut into 1-cm pieces. Intestinal tissues were transferred into HBSS buffer (Ca/Mg free) supplemented with 5% FBS, 5 mm EDTA, 10 mm HEPES (15630080; Gibco), and 0.15 mg/mL (1 mm) DTT (DTT001.05; BioShop). Then, samples were shaken for 10 min at 37°C at 200 rpm and filtered through a 100-µm strainer to remove the intestinal epithelial layer. The remaining tissue was minced and incubated in a digestion buffer containing RPMI 1640 complete medium (11875135; Gibco), 5% FBS, 0.2 U/mL Liberase (5401020001; Roche), 5 U/mL DNase I (3000 kU/mg; DRB001.1; BioShop), 100 IU/mL penicillin, and 100 µg/mL streptomycin (15140122; Gibco) for 40 min at 37°C with shaking at 150 rpm. Cell suspensions were filtered through a 100-µm strainer and centrifuged at 2000 rpm. Obtained cells were washed with PBS and used as colonic lamina propria for flow cytometry.

MLNs were harvested and put in a plate with cold PBS. Mesentery or fat was carefully removed. MLNs were gently dissociated with the plunger of a 3-mL syringe and washed with 10 mL RPMI 1,640. The cell suspension was passed through a 100-µm filter. The obtained supernatant was centrifuged at 400 × *g* for 5 min at 4°C and washed once with PBS. Pellet cells were used for flow cytometry.

### *In vitro* Th17 differentiation assay

Naïve CD4^+^ (CD4^+^ CD25^−^CD62L^+^ CD44^−^) T cells were isolated from B6 mouse spleen by cell sorting using BD FACSAria III Cell Sorter (BD Biosciences). Then, 24-well-plates were precoated with 500 μL PBS containing 2 μg/mL anti-mouse CD3 (100208; BioLegend) and 1 μg/mL anti-mouse CD28 (102112; BioLegend) overnight. In total, 0.5–1 × 10^5^ cells/well in 1.0 mL of T cell media (RPMI supplemented with 10% FBS, 55 mm 2-mercaptoethanol, and 1% Strep/Pen) were incubated in the plates for 72 h. For Th17 differentiation, 0.5 ng/mL TGF-β1 (100–21; PeproTech) and 10 ng/mL IL-6 (216-16; PeproTech) were added to the medium. For metabolite treatment, 50 μL of 20 mm nicotinic acid in 1 M NaOH or 1 M NaOH vehicle was added to the culture medium at the beginning of the experiment; 50 μL of 20 mm nicotinamide in water or water vehicle was similarly set up. Cells were harvested, and intracellular staining for IL-17A and IL-22 by flow cytometry on day 3.

### Flow cytometry

For intracellular staining, the instructions of BD Biosciences Cytofix/Cytoperm kit (554714 and 562574) for cytokines/transcription factors were followed. The cells were first stimulated with 50 ng/mL phorbol 12-myristate 13-acetate (PMA; P8139; Sigma-Aldrich), 500 ng/mL ionomycin (I0634; Sigma-Aldrich), and 10 mg/mL Brefeldin A (BFA; 420601; BioLegend) in complete RPMI media with 10% FBS at 37°C for 5 h. The cells were blocked for nonspecific binding with Fc block (553142; BD Biosciences) for 30 min.

All antibodies were diluted at 1:200 unless mentioned otherwise.

For surface staining, the cells were incubated on ice for 1 h (all antibodies are from BioLegend unless noted otherwise) with antibodies against CD45 (clone 30-F11); CD3 (clone 17A2); CD4 (clone GK1.5); TCR γδ (clone GL3); CD127 (clone A7R34); NKp46 (clone 29A1.4); and lineage cell markers, including CD11b (clone M1/70), TER-119 (clone TER-119), CD11c (clone N418), CD19 (clone 1D3/CD19), CD3 (clone 500A2), and Ly6G (RB6-8C5). Then, the cells were permeabilized and fixed in 100 µL of Fixation/Permeabilization solution (BD Biosciences) at 4°C for 20 min; washed twice in Perm/Wash buffer (BD Biosciences); and stained for intracellular cytokines with the following antibodies: IL-22 (clone Poly5164), IL-17A (clone TC11-18H10.1), and RORγt (clone Q31–378; BD Biosciences) at 4°C overnight. The cells were washed twice in Perm/Wash buffer, resuspended in a FACS buffer (1% FBS, 0.01% sodium azide, PBS), and used for flow cytometry. Data were collected with a BD LSR Fortessa and analyzed with FlowJo software.

For IgA detection from colonic lamina propria, cells were stained with surface markers, including CD45 (clone 30-F11), CD3 (clone 17A2), CD4 (clone GK1.5), CD138 (clone 281–2), and IgA (clone mA-6E1; Invitrogen).

### Quantification of IgA binding to cultured fungi in feces

Fecal samples were resuspended in sterile, cold PBS to a concentration of 200 mg/mL. Samples were broken up with a pipette tip, vortexed for 1–2 min, and spun to yield homogenates for IgA quantification.

In total, 500 μL to 1 mL of fecal homogenates were filtered through a 100-μm filter into a 50-mL conical tube. Filters were rinsed with 10 mL cold PBS and spun at 4,000 rpm for 5 min. Supernatants were discarded. Pellets were resuspended in 10 mL PBS and spun at 4,000 rpm for 5 min. Supernatants were discarded, and pellets were vortexed in residual PBS by vortexing. Then, each sample was mixed with 200 μL of 10% FBS (v/v) in PBS and incubated on ice for 10 min. Pellets were stained with IgA antibody or Rat IgG1 kappa isotype control (12-4301-81; Invitrogen) for 1 h, and washed once before analysis on the BD LSR Fortessa. To detect *C. tropicalis*, we used strains tagged with GFP or RFP.

### Immunofluorescence staining for epithelial cell proliferation

Mice were euthanized. Colon tissues were collected immediately, embedded in O.C.T Tissue-Tek (62550; Sakura Finetek), and cut into 8-µm sections on saline-coated glass slides (5116; MUTO). Slides were fixed with 4% paraformaldehyde for 20 min and permeabilized with 0.1% Triton X-100 for 10 min. The slides were blocked with 2% bovine serum albumin (ALB001; BioShop) with 0.1% Tween 20 for 1 h at room temperature and incubated with primary antibodies against Ki-67 (1:1000; ab15580; Abcam) and Ep-CAM (1:250; 118201; BioLegend) in blocking buffer at 4°C overnight. Then, the slides were incubated for 1 h at room temperature with secondary antibodies, including Alexa Fluor 488 donkey anti-rat IgG (H + L) (1:500; A-21208; Invitrogen) and Alexa Fluor 568 donkey anti-rabbit IgG (H + L) (1:500; A10042; Invitrogen). Hoechst 33,342 was used for nuclei staining (1:1,000; H3570; Invitrogen) for 5 min at room temperature. The slides were mounted with mounting medium (S3023; Dako) and covered with a coverslip. Images at 200× magnification were captured with a Zeiss microscope (Observer Z1, Zeiss).

### Intestinal permeability test

Mice were fasted for 4 h before obtaining 150 µL of 10 mg/mL FITC-dextran (FD4; Sigma-Aldrich). Blood was collected by cardiac puncture and added to 15% citrate-dextrose solution (C3821; Sigma-Aldrich). Samples were centrifuged at 10,000 rpm for 10 min at 4°C. Plasma was diluted 1:10 and 1:100 in PBS, and fluorescence was measured at 530 and 485 nm with SpectraMax i3× (Molecular Devices). Results were presented as relative fluorescence units.

### Filamentation assay in vitro

*C. tropicalis* strain was incubated overnight at 30°C in YPD broth. 10^6^ CFUs of each strain (MYA-3404 and YM990490) were added to 500 μL RPMI + 10% FBS at 37°C. About 10 μL of supernatant was collected at the different time points 2-, 4-, 6-, 10- and 24-h post incubation, then fixed with 5 μL 1% formaldehyde and imaged with brightfield microscopy.

### Nicotinamidase activity

Nicotinamidase activity was assayed based on the absorbance method, as described.^48^ In brief, *C. tropicalis* (MYA-304 and YM990490 strains) was cultivated and prepared as stated above. Fungal pellets (2 × 10^6^) were resuspended in 300 μL of 2 mm nicotinamide (N0636; Sigma-Aldrich) and 0.75% sodium nitroprusside (SNP; 228710; Sigma) for 4 and 6 h in 96 well-plates. Plates were centrifuged at 2000 rpm for 3 min, and 150 μL of supernatant was collected to measure absorbance at 395 nm. The remaining samples were diluted serially for fungal culture with YPD agar.

Enzyme activity (μmol/min) = consumed nicotinamide/reaction time (min) × sample volume (mL)

### Metabolite analysis of fecal samples

+ Sample preparation: In total, 35 mg of lyophilized fecal pellets were dissolved in 875 μL of ice-cold water, vortexed for 5 min at 1,000 rpm, ultrasonicated for 20 min at room temperature, and centrifuged at 15,000 × *g* for 15 min at 4°C. The supernatants were collected. The pellets were further extracted with 875 μL of ice-cold MeOH using the same procedure. The supernatants obtained from both extraction procedures were pooled, passed through a 0.22-μm filter, and stored at −20°C for UHPLC-Orbitrap analysis.

+ LC-MS analysis: Metabolomics analysis was performed using a Dionex Ultimate 3000 UHPLC system with quaternary pumps coupled with a Q Exactive Plus hybrid quadrupole-Orbitrap mass spectrometer (Thermo Fisher Scientific). MS spectra were acquired in positive ionization mode via top-5 data-dependent acquisition, with a spectral resolution of 70,000 for the MS spectrum and a scan range of 80 − 1,200 *m/z*. Data-dependent MS/MS (ddMS2) spectra were acquired with a spectral resolution of 17,500; isolation window of 1.5 m/z; stepped normalized CE of 15, 30, 45; and dynamic exclusion of 15.0 s. The AGC targets established for full MS and ddMS2 were 3e6 and 5e5, correspondingly.

+ Data processing: Compound Discoverer 3.3.0 (Thermo Fisher Scientific) was used to process raw metabolomics data. The workflow included peak extraction, retention time alignment, metabolite identification, QC correlation, gap status, and background subtraction. For peak extraction and retention time alignment, mass tolerance was set at 5 ppm1, maximum shift of 0.2 min using the adaptive curve alignment model, intensity tolerance of 30% for isotope search, signal-to-noise threshold of 3, minimum peak intensity of 1,000,000, and a minimum of seven scans per peak. In order to identify metabolites, the identified peaks were annotated with their similarity to authentic metabolite spectra in the database or reference spectra obtained from online sources (mzCloud, metabolika, Chemspider). Compound Discoverer computed the similarity by considering isotope similarity, precursor mass error (<5 ppm), and fragmentation spectra, where applicable.

+ Data analysis: The area was normalized by an internal standard to eliminate the effect of matrices from sample variation. To compare the two groups, a two-tailed Student’s *t*-test was employed. To minimize false-positive discovery, we evaluated all *p* values derived from the two-tailed Student’s *t*-test using the false discovery rate (FDR) method. A threshold for FDR was established at <0.05. Statistical analysis and fold change were calculated using Python 3.11. Volcano plots were generated using Microsoft Excel. MetaboAnalyst 5.0, a web-based metabolomics data processing tool, was used to create heatmaps and perform principal component analysis, partial least squares discriminant analysis, and pathway analysis.

### Quantification and statistical analysis

All data are shown as the mean ± SD. Statistical significance among groups was calculated using one- or two-way analysis of variance, followed by Tukey’s multiple comparisons test (comparison of ≥3 groups) or unpaired *t*-test (for two groups), using GraphPad Prism version 9.0.2 (GraphPad Software). *p* values ≤ 0.05 were considered statistically significant and were denoted as *p < 0.05, **p < 0.01, ***p < 0.001, and ****p < 0.0001.

## Supplementary Material

Supplementary_file.docx
