# Supplementary material for: Candida tropicalis-derived vitamin B3 exerts protective effects against intestinal inflammation by promoting IL-17A/IL-22-dependent epithelial barrier function
Source: Gut Microbes. 2024 Oct 27;16(1):2416922. doi: 10.1080/19490976.2024.2416922 (PMC11524206; doi:10.1080/19490976.2024.2416922)
Supplement: Supplementary_file.docx [file KGMI_A_2416922_SM4307.docx]

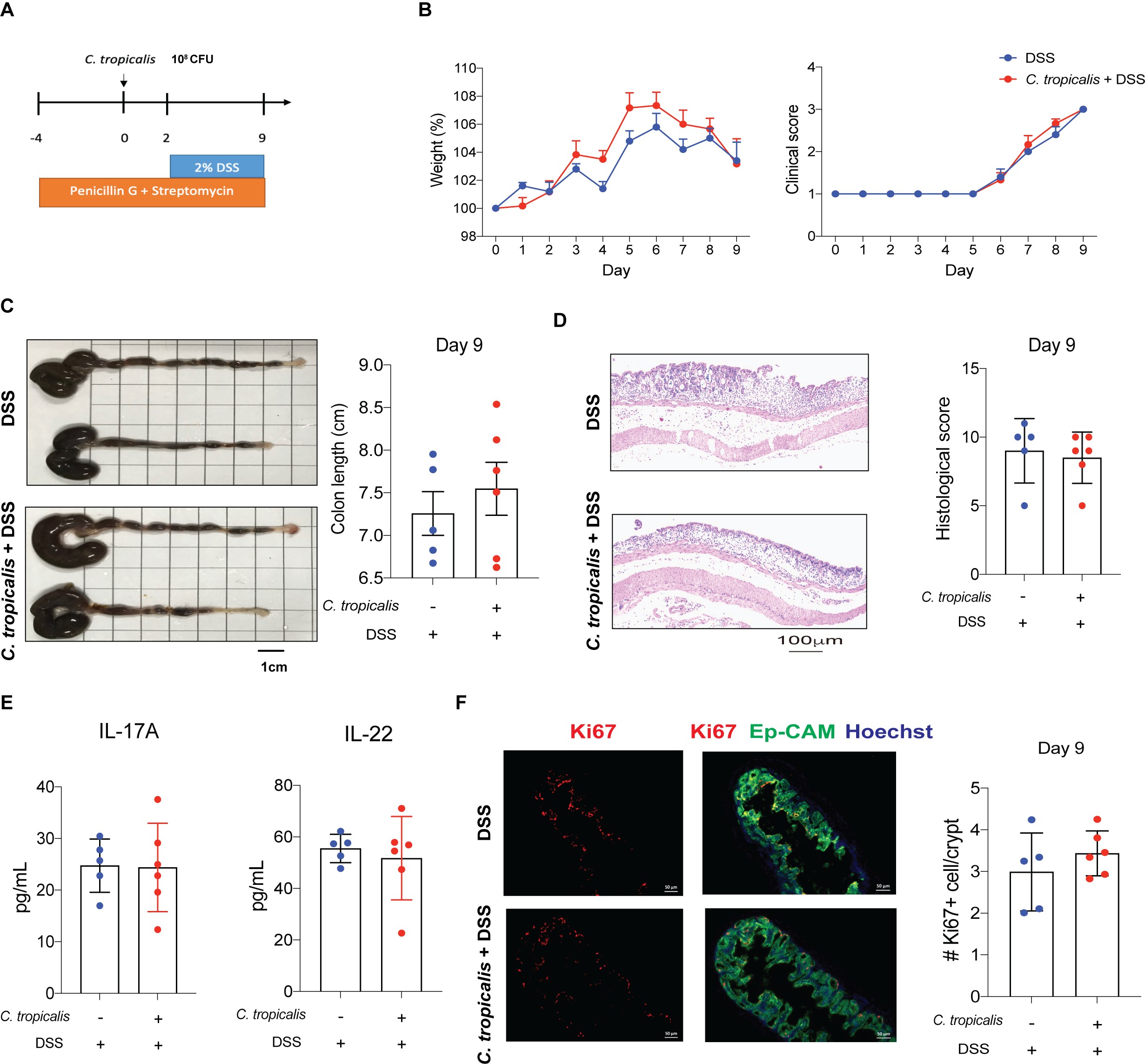


# Figure S1. *Candida tropicalis* supplementation has no discernible impact on the severity of colitis during the induction phase

1. Experimental design. Mice were induced colitis with 2% DSS for 7 days.
2. Weight loss and total clinical scores of the control and *C. tropicalis* groups. n = 5 - 6 per group of two experiments.
3. Representative image of colon and colon length between two groups. n = 5 - 6 per group of two experiments.
4. Representative image of H&E staining and histological scores between two groups. n = 5 – 6 per group of two experiments.
5. IL-17A and IL-22 levels from distal colonic homogenates of two groups. n = 5 – 6 per group of two experiments.
6. Representative immunofluorescent images of epithelial cell proliferation with Ki-67 antibody (cell proliferation) and Ep-CAM (epithelial cells). Quantify the Ki-67^+^ cells per crypt between control and

*C. tropicalis* groups. n = 5 - 6 per group of two experiments.

Analyzed with two-way ANOVA (Fig. S1B) and t-test (Fig. S1C-F).


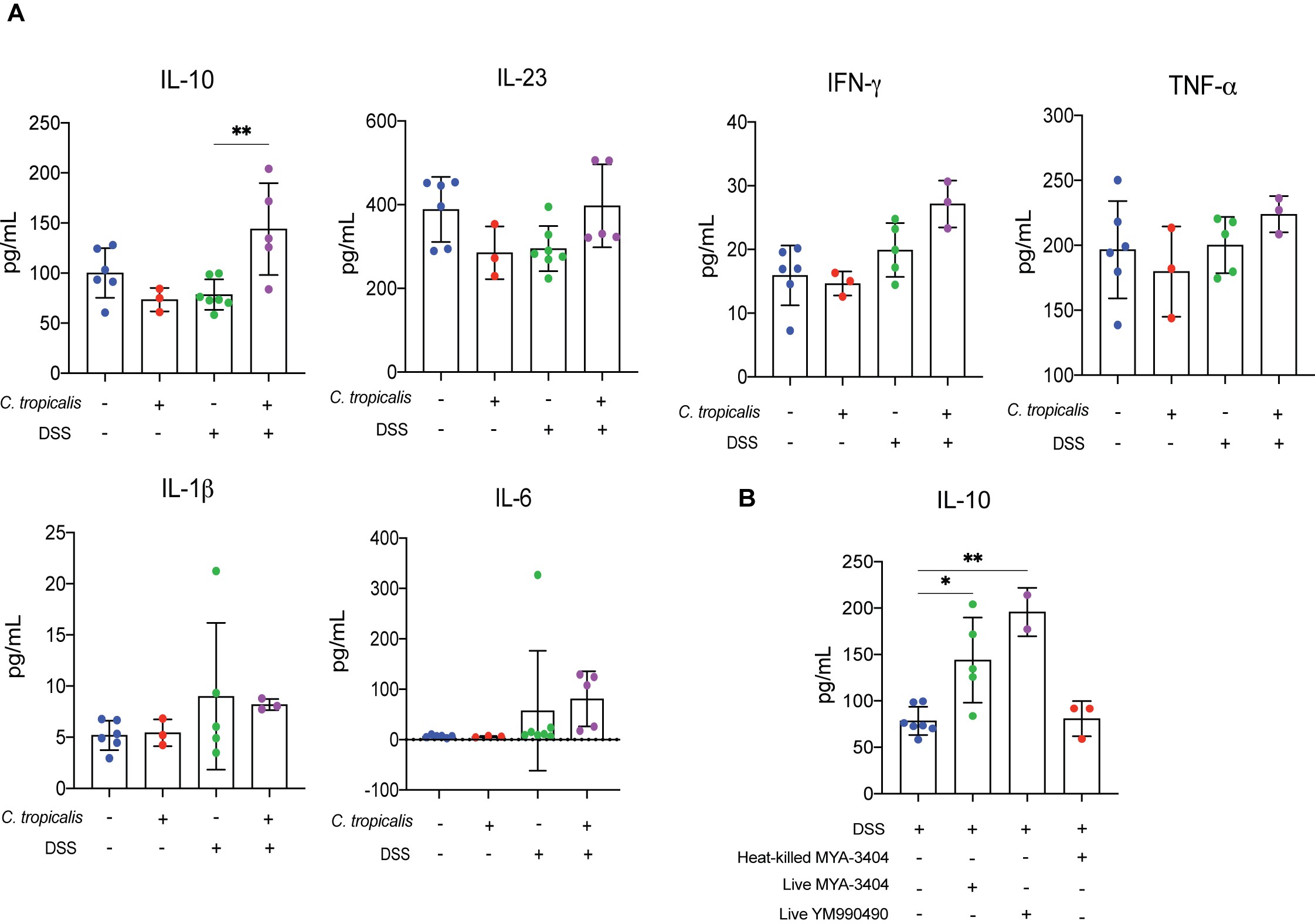


# Figure S2. Cytokine production of *C. tropicalis* colonization in the intestine

1. IL-10, IL-23, IFN-γ, TNF-α, IL-1β, and IL-6 levels are determined in the colonic homogenates of control and DSS-induced groups. The results are pooled data from two separate experiments. *n* = 3 – 7 per group.
2. IL-10 from the distal colon of different strains MYA-3404 and YM990490. *n* = 2 – 15 per group from three experiments.

Statistical analyses were performed with one-way ANOVA. *, *p*<0.05, **, *p*<0.01.


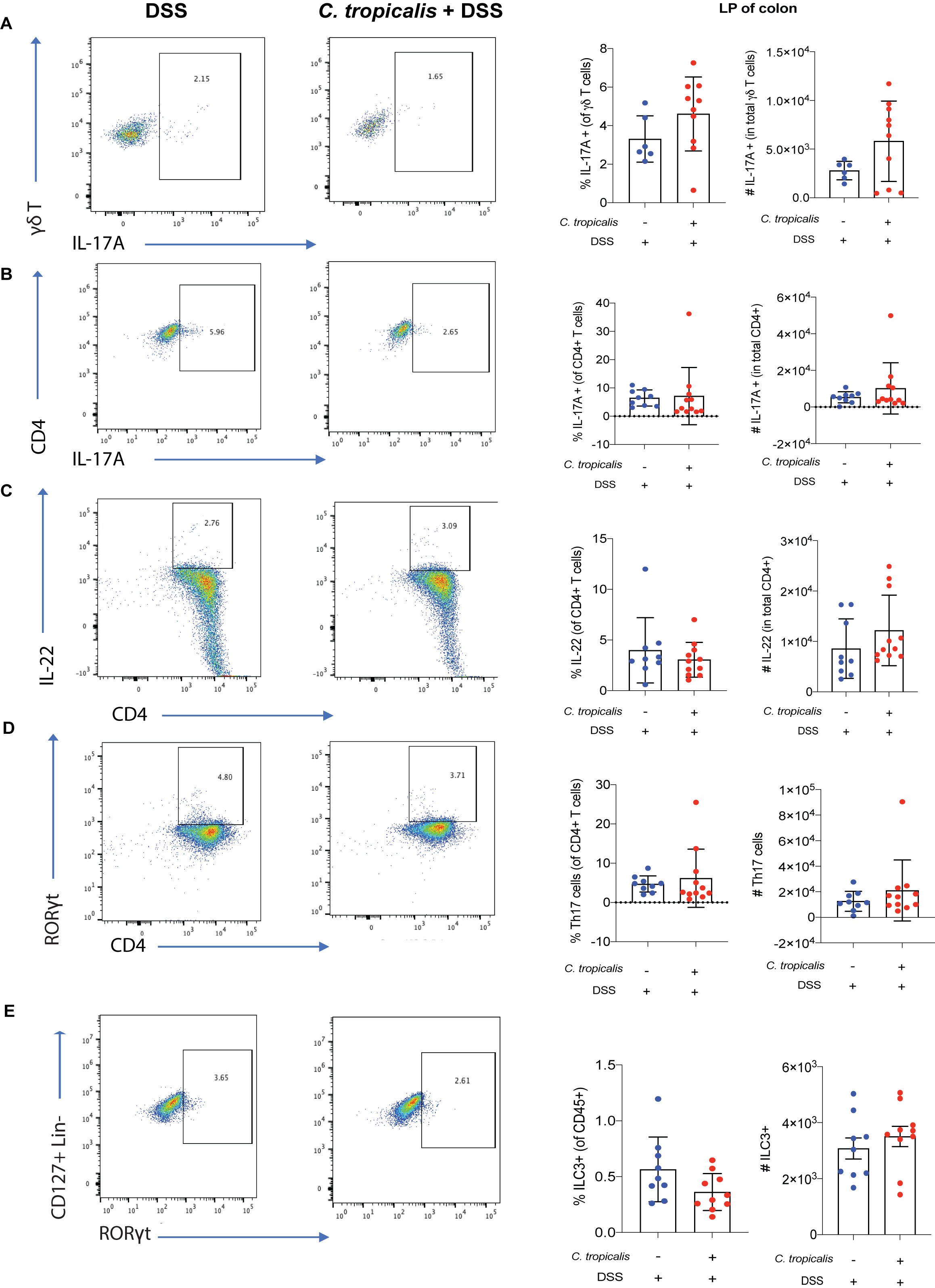


# Figure S3. *C. tropicalis* colonization reveals no immune cell shift in colonic lamina propria

1. Representative flow cytometry plots and quantification of IL-17A^+^ cells from γδ T cells in LP of control and *C. tropicalis* groups. n = 6 in control, n = 10 in *C. tropicalis* group of four experiments.
2. Representative flow cytometry plots and quantification of IL-17A^+^ cells from CD4^+^ T cells in LP of control and *C. tropicalis* groups. n = 10 in control, n = 13 in *C. tropicalis* group of four experiments.
3. Representative flow cytometry plots and quantification of IL-22^+^ cells from CD4^+^ T cells in LP of control and *C. tropicalis* groups. n = 9 in control, n = 12 in *C. tropicalis* group of four experiments.
4. Representative flow cytometry plots and quantification of Th17 cells from CD4^+^ T cells in LP of control and *C. tropicalis* groups. n = 9 in control, n = 12 in *C. tropicalis* group of four experiments.
5. Representative flow cytometry plots and quantification of ILC3 cells from CD45^+^ cells in LP of control and *C. tropicalis* groups. n = 9 in control, n = 10 in *C. tropicalis* group of four experiments.

Statistical analyses were performed with t-test. Lamina propria (LP).


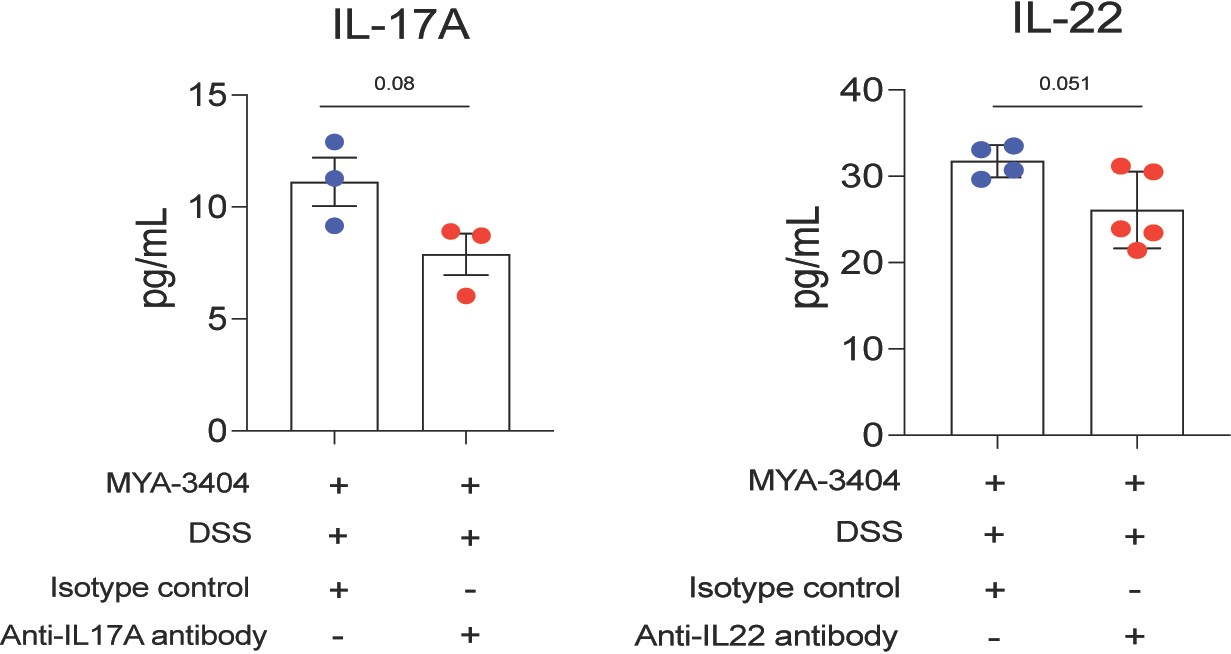


# Figure S4. Neutralizing antibodies decreased cytokines IL-17A and IL-22 expression in the mouse colons

Levels of IL-17A and IL-22 from the distal colon was measured by ELISA. n = 3 - 5 per group. Statistical analyses were performed with t-test.


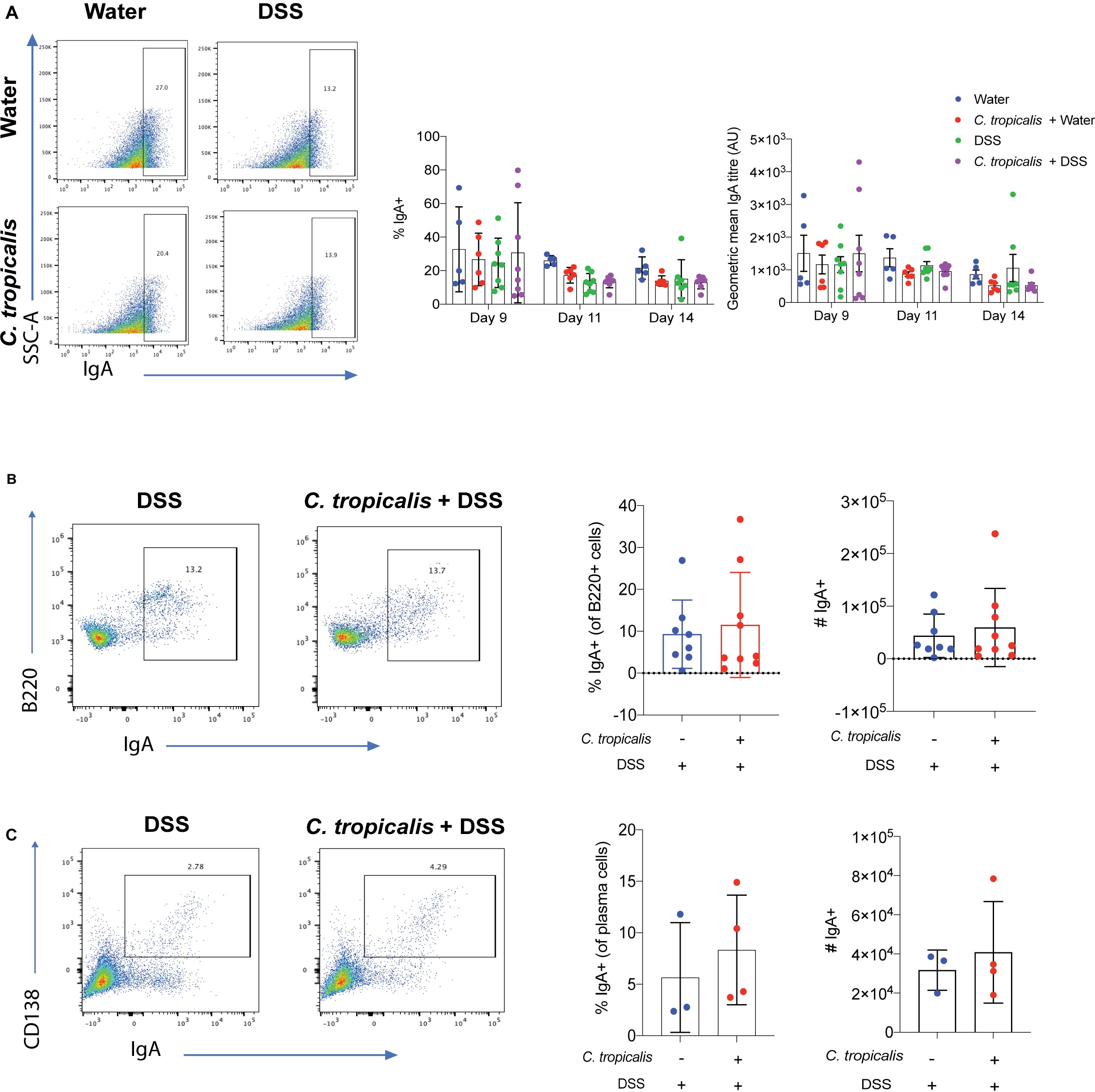


# Figure S5. sIgA is not involved in the immune response to *C. tropicalis* colonization in the intestine

1. Representative flow cytometry plots and quantification of IgA in fecal samples on days 9, 11, and 14. n = 6 in non-DSS groups, n = 9 in DSS groups of two experiments.
2. Representative flow cytometry plots and quantification of IgA^+^ of B cells in LP of the colon. n = 8 in control, n = 9 in *C. tropicalis* group of two experiments.
3. Representative flow cytometry plots and quantification of IgA^+^ of plasma cells in LP of colon. n = 3 in control, n = 4 in *C. tropicalis* group of two experiments.

Statistical analyses were performed with two-way ANOVA (Fig. S5A) and t-test (Fig. S5B-C).


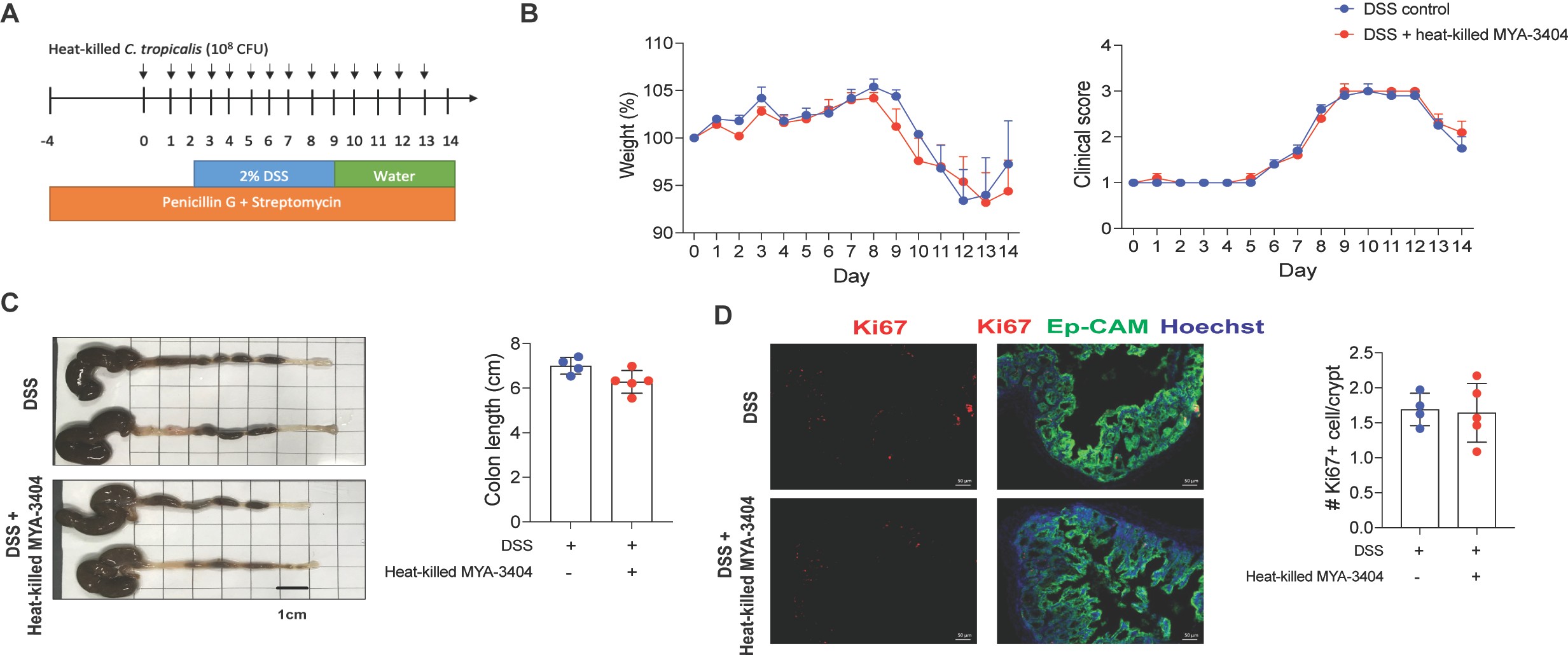


# Figure S6. Heat-killed MYA-3404 group has minimal impact on colitis severity

1. Experimental design. Mice were daily gavaged with PBS or heat-killed MYA-3404 during the experiment.
2. Body weight loss and total clinical scores between two groups. n = 4 - 5 per group.
3. Representative images and colon length of two groups. n = 4 - 5 per group.
4. Representative immunofluorescent images and quantify the Ki-67^+^ cells per crypt between two groups. n = 4 - 5 per group of two experiments.

Statistical analyses were performed with two-way ANOVA (Fig. S6B) and t-test (Fig. S6C-D).


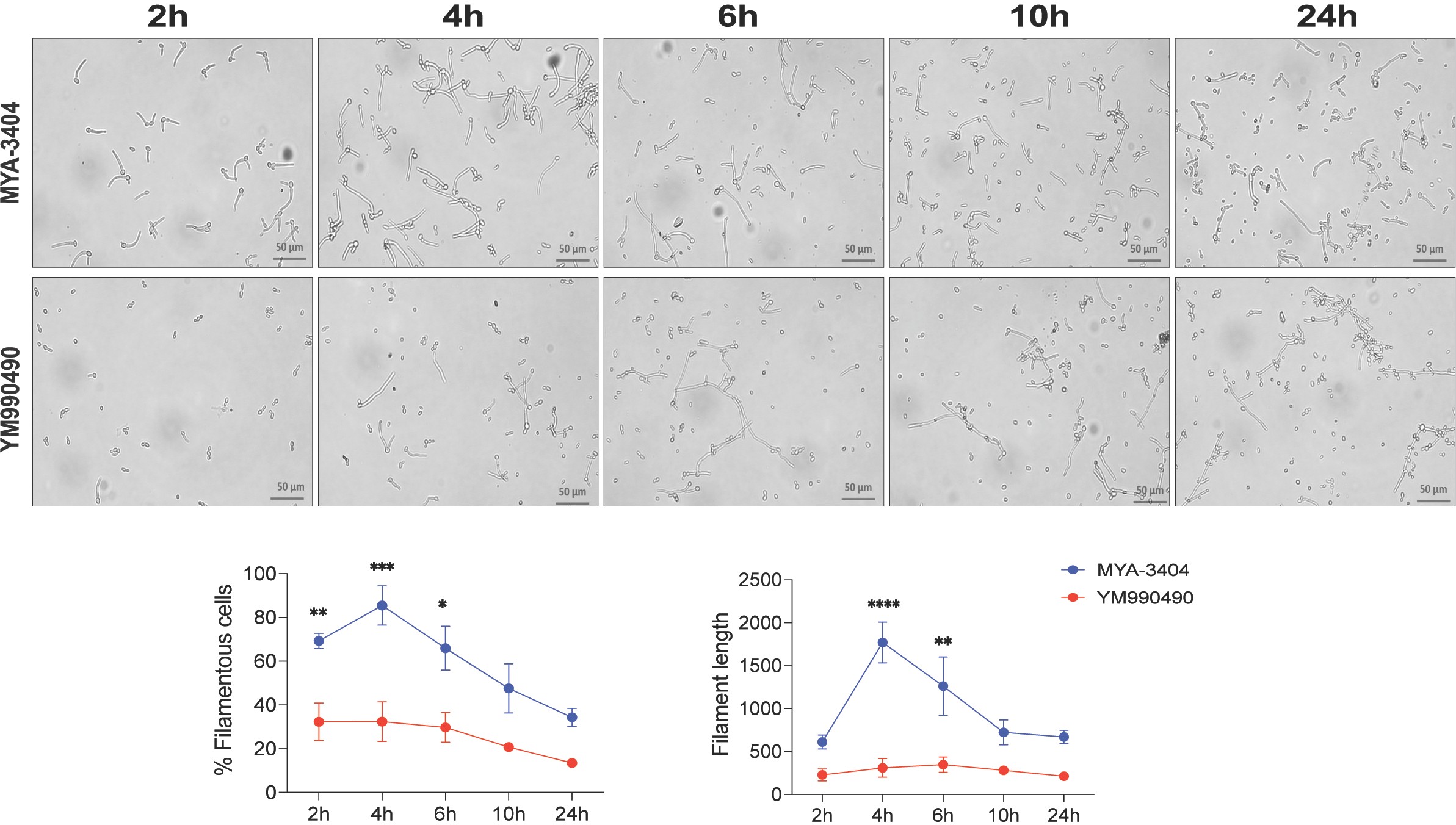


# Figure S7. Both MYA-3404 and YM990490 are capable of inducing hyphal morphology.

Representative bright-field images of fungal cells following hyphal induction in RPMI with 10% FBS at 37^o^C at different times. Percentage of filamentous cells and the length of filaments between two strains.

Statistical analyses were performed with two-way ANOVA. **p* < 0.05, ***p* < 0.01, ****p* < 0.001, and

*****p* < 0.0001.


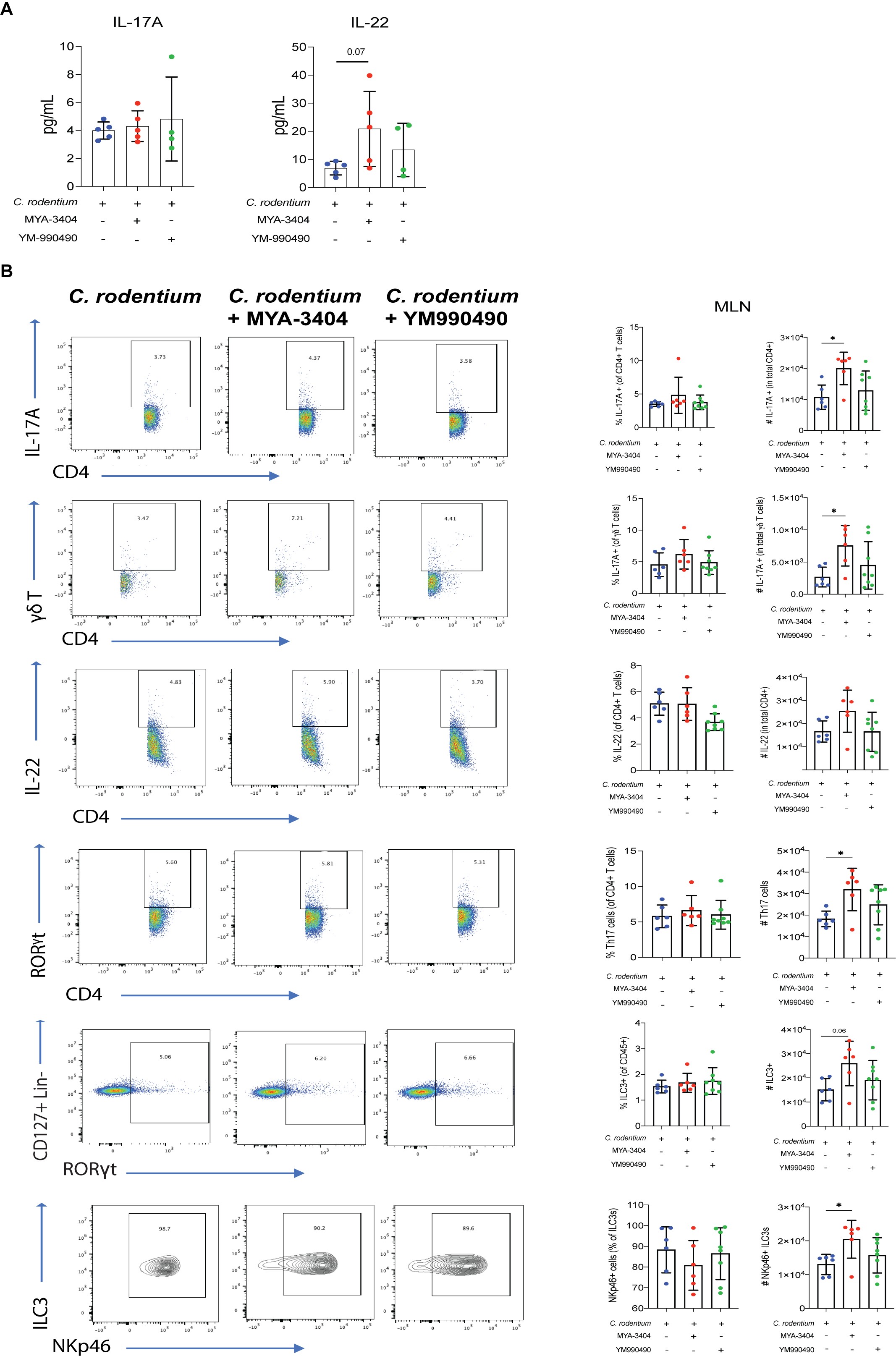


# Figure S8. Cytokines and immune cell changes in *C. rodentium* infection

1. Level of IL-22 and IL-17A from the distal colon of three groups. n = 4 – 5 per group from one experiment.
2. Representative flow cytometry plots and quantification (percentage and total counts) of different immune cells (IL-17A^+^ CD4^+^, IL-17A^+^ γδ T, IL-22^+^ CD4^+^, Th17, ILC3, and NKp46^+^ ILC3) from MLN.

n = 6 – 8 per group from two experiments.

Statistical analyses were performed with one-way ANOVA. *, *p*<0.05.


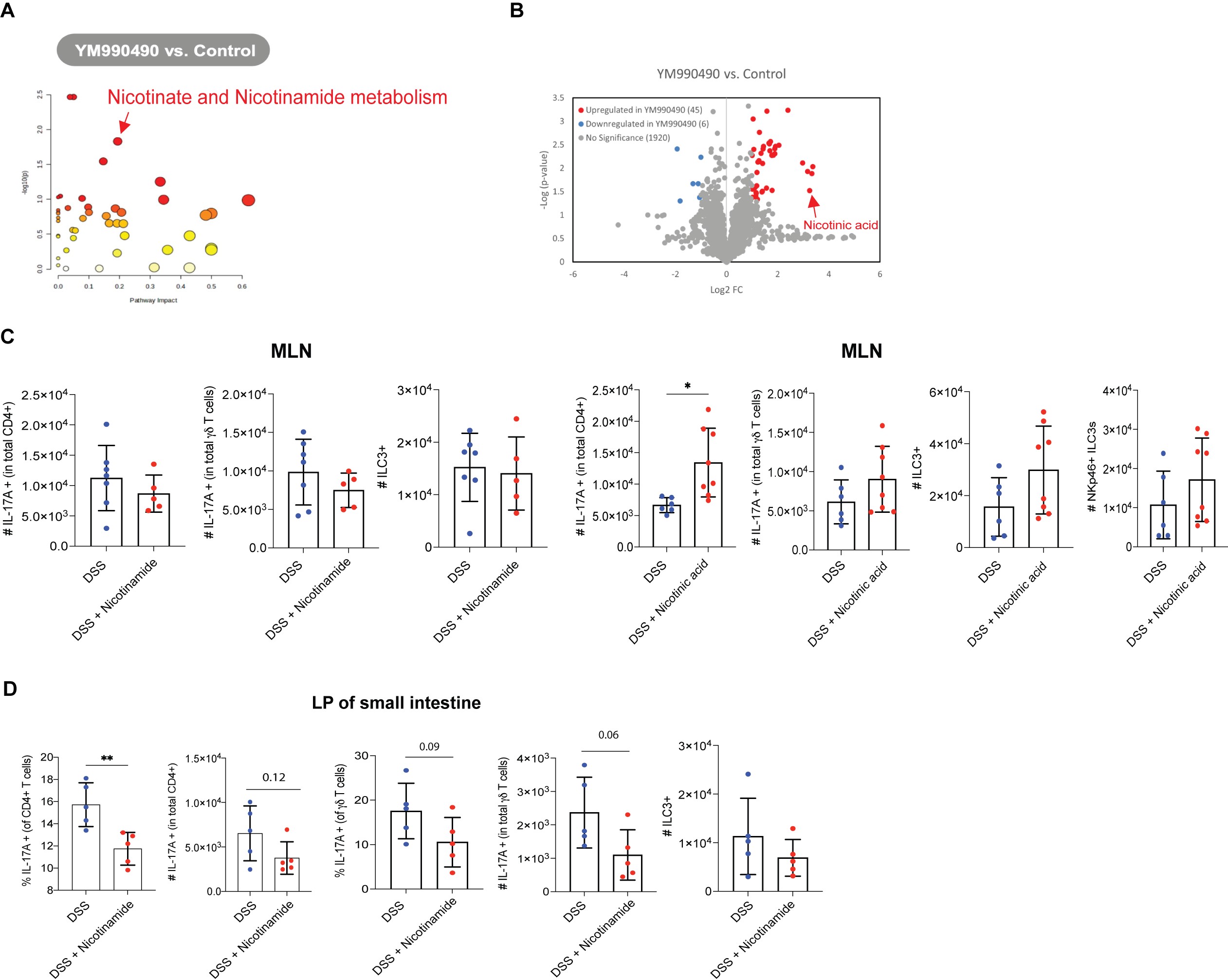


# Figure S9. Metabolic profile of YM990490 group and Immune cell changes in vitamin B3 supplementation

1. Pathway analysis of differentially expressed metabolites was analyzed with MetaboAnalyst between YM990490 and control groups.
2. Volcano plot showing the fold-change of metabolite quantity between YM990490 and control groups.

Fold change > 2 or < -2, and p-value < 0.05 were highlighted in red and blue, respectively.

1. Quantification of immune cells (IL-17A^+^ CD4^+^, IL-17A^+^ γδ T, and ILC3s) isolated from MLN of control and nicotinamide/nicotinic acid groups. n = 5 – 8 per group from two experiments.
2. Quantification of immune cells (IL-17A^+^ CD4^+^, IL-17A^+^ γδ T and ILC3) from the lamina propria (LP) from the small intestine of control and nicotinamide groups. n = 5 per group from one experiment.

Statistical analyses were performed with t-test (Fig. S9C-D).
